# Application of mathematical modelling to inform national malaria intervention planning in Nigeria

**DOI:** 10.1186/s12936-023-04563-w

**Published:** 2023-04-26

**Authors:** Ifeoma D. Ozodiegwu, Monique Ambrose, Beatriz Galatas, Manuela Runge, Aadrita Nandi, Kamaldeen Okuneye, Neena Parveen Dhanoa, Ibrahim Maikore, Perpetua Uhomoibhi, Caitlin Bever, Abdisalan Noor, Jaline Gerardin

**Affiliations:** 1 Department of Preventive Medicine and Institute for Global Health, Northwestern University, Chicago, IL USA; 2 Institute for Disease Modeling, Seattle, WA USA; 3 Global Malaria Programme, World Health Organization, Geneva, Switzerland; 4 Weinberg College of Arts and Sciences, Northwestern University, Evanston, IL USA; 5 National Malaria Elimination Programme, Abuja, Nigeria

**Keywords:** Malaria, Mathematical modeling, Subnational tailoring of interventions, Stratification, Intervention targeting, National strategic planning, Transmission models, Impact predictions

## Abstract

**Background:**

For their 2021–2025 National Malaria Strategic Plan (NMSP), Nigeria’s National Malaria Elimination Programme (NMEP), in partnership with the World Health Organization (WHO), developed a targeted approach to intervention deployment at the local government area (LGA) level as part of the High Burden to High Impact response. Mathematical models of malaria transmission were used to predict the impact of proposed intervention strategies on malaria burden.

**Methods:**

An agent-based model of *Plasmodium falciparum* transmission was used to simulate malaria morbidity and mortality in Nigeria’s 774 LGAs under four possible intervention strategies from 2020 to 2030. The scenarios represented the previously implemented plan (business-as-usual), the NMSP at an 80% or higher coverage level and two prioritized plans according to the resources available to Nigeria. LGAs were clustered into 22 epidemiological archetypes using monthly rainfall, temperature suitability index, vector abundance, pre-2010 parasite prevalence, and pre-2010 vector control coverage. Routine incidence data were used to parameterize seasonality in each archetype. Each LGA’s baseline malaria transmission intensity was calibrated to parasite prevalence in children under the age of five years measured in the 2010 Malaria Indicator Survey (MIS). Intervention coverage in the 2010–2019 period was obtained from the Demographic and Health Survey, MIS, the NMEP, and post-campaign surveys.

**Results:**

Pursuing a business-as-usual strategy was projected to result in a 5% and 9% increase in malaria incidence in 2025 and 2030 compared with 2020, while deaths were projected to remain unchanged by 2030. The greatest intervention impact was associated with the NMSP scenario with 80% or greater coverage of standard interventions coupled with intermittent preventive treatment in infants and extension of seasonal malaria chemoprevention (SMC) to 404 LGAs, compared to 80 LGAs in 2019. The budget-prioritized scenario with SMC expansion to 310 LGAs, high bed net coverage with new formulations, and increase in effective case management rate at the same pace as historical levels was adopted as an adequate alternative for the resources available.

**Conclusions:**

Dynamical models can be applied for relative assessment of the impact of intervention scenarios but improved subnational data collection systems are required to allow increased confidence in predictions at sub-national level.

**Supplementary Information:**

The online version contains supplementary material available at 10.1186/s12936-023-04563-w.

## Background

Although Nigeria has made substantial progress in malaria control, it continues to bear a disproportionate share of the global malaria burden, accounting for an estimated 27% and 23% of all malaria cases and deaths in 2019, respectively [1]. Previously accelerated declines in incidence and deaths have plateaued, and at the current pace of progress, it is unlikely that the Global Technical Strategy (GTS) targets of reducing malaria incidence and mortality by at least 75% and 90% by 2025 and 2030 will be met [1, 2]. Early signals of these concerning trends prompted the World Health Organization (WHO) and the RBM Partnership to End Malaria to launch the ‘High Burden to High Impact’ (HBHI) initiative, a country-led approach [3]. In its first phase in 2019–20, HBHI supported 11 high-burden countries, including Nigeria, to accelerate progress towards achieving the GTS milestones. Countries participating in the HBHI initiative aim to maximize the impact of malaria control tools by deploying them in areas where they will be most effective. The approach consists of four response elements: (1) promotion of political will to reduce malaria deaths, (2) the use of strategic information to drive impact, (3) the development of better guidance, policies, and strategies, and (4) a coordinated national malaria response. As part of the second HBHI response element, Nigeria’s National Malaria Elimination Programme (NMEP) developed a targeted approach for intervention deployment at the local government level for the 2021–2025 National Malaria Strategic Plan (NMSP). Mathematical modellers were recruited to create an analytical framework for predicting the impact of the NMEP’s proposed intervention strategy on malaria morbidity and mortality in each of Nigeria’s 774 local government areas (LGAs).

Spatial variation in malaria transmission intensity, seasonality, and other contextual factors associated with malaria risk were key factors that motivated tailoring of interventions to the LGA-level. Malaria transmission intensity in Nigeria varies from areas of hypo-endemicity to hyper-holo endemicity [4–8], making a one-size-fits-all approach to intervention deployment suboptimal. For example, geostatistical models of parasite prevalence and evidence from the 2018 Demographic and Health Survey (DHS) show concentration of the childhood malaria burden in the north-west, south-west and north-central states, and areas of low and medium endemicity within the same state boundary [4]. At a finer scale, spatial variation in malaria risk within and across urban and rural areas justified the need for targeting malaria interventions [4, 6, 9]. Moreover, inter- and intra-state variation in the start and end of the transmission season should be considered in allocating seasonal malaria chemoprevention, typically administered in highly seasonal or epidemic regions [10]. Access to care and care-seeking propensity toward public versus private treatment providers also varies [11, 12]. Considering these factors, the NMEP stratified interventions by LGA, arriving at a resource-agnostic intervention mix comprised of effective case management for malaria, treated bed nets with pyrethroid piperonyl butoxide active ingredient, intermittent preventive treatment in pregnancy, seasonal malaria chemoprevention, and intermittent preventive treatment in infants; with 80% or higher coverage.

Widening shortfalls in funding for malaria programmes emphasize the need for optimal allocation of interventions to maximize impact given limited resources [1]. Since insufficient funds were available to implement the full NMSP, two prioritized plans were developed to fit within a specified resource envelope. Comparisons of the resource-agnostic plan and prioritized plans in the context of limited resources were crucial to understand each plan’s impact on malaria burden and to inform the selection of the final intervention mix and grant applications to the Global Fund to Fight AIDS, TB and Malaria.

Mathematical frameworks can be used to develop models of malaria transmission, which integrate historical data on transmission intensity, seasonality, and intervention coverage at the desired spatial scale with expert opinion. These models can be simulated forward to predict the impact of multiple intervention scenarios on malaria prevalence, incidence and mortality [13–18]. What follows is a description of how EMOD, a well-elaborated mathematical model of malaria transmission [19–21], was applied to support national malaria strategic planning and resource prioritization by the NMEP by making LGA-level impact predictions for four intervention scenarios.

## Methods

### Overview

**Fig. 1:**
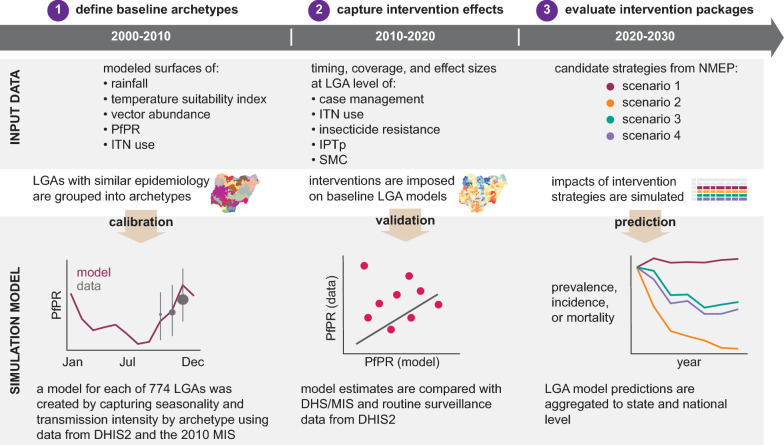
Methodological overview for this study with illustrations of the approach

A three-step process was used to generate LGA-level predictions of potential national strategic plans (Fig. 1). The first step was to capture the intrinsic potential of each LGA to support malaria transmission in a baseline period before most interventions were scaled up nationwide. Data and geospatial modelled surfaces were used to group LGAs into epidemiological archetypes. For each archetype, baseline malaria transmission was calibrated to 2014–18 monthly malaria case data and prevalence data collected from children under the age of five years in the 2010 malaria indicator surveys to capture seasonality and transmission intensity, respectively. Next, Nigeria’s intervention history from 2010 to 2020 at the LGA level was imposed on the baseline models to generate 774 LGA-level models up through 2020. Last, various future intervention strategies were applied to the LGA models, and the impact of each intervention strategy on prevalence, incidence, and mortality was assessed. The following sections summarize the study methods. More details are provided in Additional file 1.

### Clustering LGAs with similar baseline transmission patterns to generate epidemiological archetypes

**Fig. 2:**
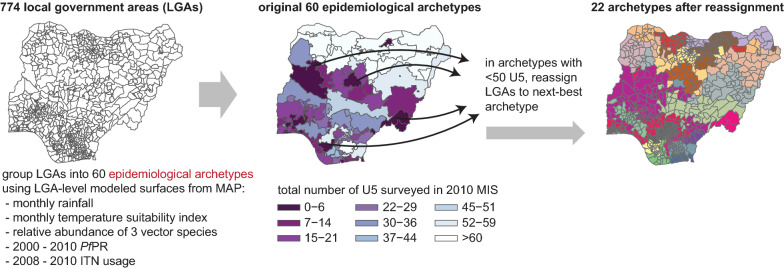
Hierarchical clustering of 774 LGAs into 22 epidemiological archetypes using monthly rainfall, monthly temperature suitability index, relative abundance of vector species, 2000–2010 *Pf*PR, and 2008–2010 ITN use rates. *Pf*PR and ITN coverage estimates for the initial classification were obtained from the Malaria Atlas Project

Calibrating baseline transmission intensity and computing intervention coverages for each LGA in the model was challenging due to the computational power required to simulate 774 LGAs, and the lack of subnational data for some LGAs. To address this problem, LGAs with similar baseline malaria transmission patterns (climatic patterns, vectors, and baseline transmission intensity) were clustered (Fig. 2). The following LGA-level features were used for clustering: average monthly rainfall [22], average monthly temperature suitability index (TSI) [23], overall relative abundance of three vector species [24], annual modelled *Plasmodium falciparum* parasite prevalence (*Pf*PR) values from the Malaria Atlas Project (MAP) [25, 26] for 2000, 2004, 2006, 2008, and 2010, and estimated ITN use between 2008 and 2010 from MAP [27]. LGAs that shared similar climatic, vector, and transmission features were grouped into 60 epidemiological archetypes using the CLARA algorithm (see Additional file 1) [28, 29]. Given that data from the 2010 Malaria Indicator Survey (MIS) was used to calibrate parasite prevalence, archetypes where the 2010 MIS sample population of children under the age of 5 years (U5) was less than 50 were reassigned to the next closest archetype (see Additional file 1). This final reassignment resulted in 22 epidemiological archetypes with between 13 to 92 LGAs per archetype. See Additional file 1 for the archetype assignment of each LGA.

### EMOD malaria transmission model

Malaria transmission and intervention impact was simulated using EMOD v2.20, an agent-based model of *P. falciparum* transmission that comprises a model of temperature-dependent vector lifecycle and vector population dynamics, coupled to a model of human disease and immunity, and intervention effects [21, 30–33]. Parasite dynamics, immune acquisition, and clinical incidence by age were previously calibrated to field data from nine study sites in sub-Saharan Africa [34]. LGA daily air temperatures were computed using centroid longitude and latitude and data from Global Surface Summary of the Day [35]. Three local vector populations were simulated—*Anopheles gambiae s.s.*, *Anopheles arabiensis,* and *Anopheles funestus—*with species-specific parameters for anthropophily [35–38] and probability of feeding indoors [39–41]. The number of mosquito bites per person (biting risk) was modelled with an exponential distribution for exposure [42] and surface area dependence by age to capture the heterogeneity of exposure between individuals within an LGA. Population size for each LGA was rescaled to 1000 to reduce computational burden, and rescaled in the post-simulation analyses. Uniform birth and death rates were used for all LGAs [43]. Modelled LGAs were not spatially connected, and 10 infections were imported into each LGA per year.

Model inputs and scripts used for running simulations and post-processing of results are publicly available (see Data Availability section), including instructions for downloading dependencies. Scripts were written using Python 3.6.0 [44] and R 4.02 [45].

### Model calibration

#### Setting model seasonality: parameterizing monthly larval habitat availability

**Fig. 3:**
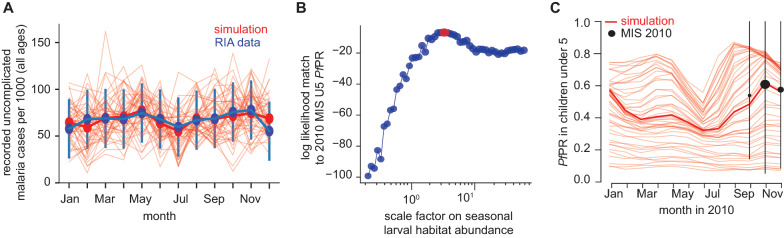
Example calibration of transmission seasonality and intensity in the Akinyele archetype. **A** Simulated seasonality of clinical malaria within the Akinyele archetype compared with rescaled RIA health facility confirmed malaria case data for years 2014–2018. Red: 50 individual stochastic realizations (thin lines) and mean (thick line) incidence of the same modelled seasonality and scaling. Blue: RIA data with 95% confidence intervals. **B** Larval habitat scale factor sampling used to match simulated *Pf*PR in the Akinyele archetype baseline model to *Pf*PR in the 2010 MIS. Red dot: best match scale factor. **C** Red: simulated U5 *Pf*PR in the Akinyele archetype. The thick red line indicates the best match while thin red lines show *Pf*PR under other larval habitat scale factors. Each line (thick and thin) is the mean of 10 stochastic realizations. Black: archetype U5 *Pf*PR from the 2010 MIS, with 95% confidence intervals

Seasonality of malaria within each archetype was calibrated to routine reporting of all-age uncomplicated malaria cases collected for a Rapid Impact Assessment (RIA) performed in 2019 by the NMEP. The RIA dataset contains quality-assured monthly confirmed plus presumed malaria cases from 917 nationally representative public health facilities in 601 LGAs in Nigeria for the period of 2014–18. Confirmed cases were diagnosed with either a rapid diagnostic test (RDT) or microscopy. All cases in each month were summed for each LGA and by year. The average number of monthly cases and 95% confidence intervals across all years were computed by LGA and summed by archetype. The monthly case series were rescaled such that the number of cases per month was roughly between 20 and 100 prior to attempting the simulation fit, since the model was well-behaved in this range of monthly treated cases and the extent to which the RIA data captured the true clinical burden of malaria in the archetype was unknown. For each of the 22 archetypes, seasonal variation in transmission intensity was captured in EMOD by selecting overall monthly *Anopheles* larval habitat availabilities such that the resulting monthly mean clinical incidence matched the shape of the monthly clinical incidence in the RIA dataset (Fig. 3A). The same seasonality was assumed for all three vector species.

#### Accounting for non-malarial fevers

Passive surveillance, such as the RIA dataset, also contains individuals who test positive for malaria but whose symptoms are caused by a non-malarial illness, such as when an individual with an asymptomatic malaria infection becomes co-infected with another pathogen. These malaria-attributed, but not malaria-caused, fevers were accounted for in the simulations by testing a random 0.38% of the simulated population daily with an HRP2-based RDT [46], approximating a baseline rate of non-malarial febrile illness. Individuals testing positive received antimalarial treatment and were added to the daily tally of malaria cases.

#### Setting baseline transmission intensity

Simulated baseline transmission intensity was evaluated using *P. falciparum* prevalence detected by microscopy with a sensitivity of 50 parasites per microliter [47]. Accurately modelling parasite prevalence for each archetype in the pre-2010 model required applying a scaling factor on the monthly vector larval habitat availability to reproduce the 2010 MIS *Pf*PR in children U5, in the presence of the observed 2010 ITN usage and case management (CM) coverage (Fig. 3B, C). *Pf*PR, ITN and CM coverage were computed from the MIS by taking a cluster-weighted averages across all LGAs within each archetype (clusters in the MIS and DHS survey are enumeration areas as defined by the census or an existing sampling frame). Since MIS data collection spanned a 2–3 month window, *Pf*PR weighted averages were computed by archetype and month of data collection.

Fifty larval habitat scale factors were sampled for each of the 22 archetypes, resulting in 1100 unique scale factor archetype combinations. For each of these combinations, a 50-year initialization phase was run to establish population immunity in the absence of interventions. For each archetype, an ITN distribution with archetype-specific coverage as observed in MIS was applied to each of the partially immune populations generated under each of the larval habitat scale factors. The U5 *Pf*PR during the months corresponding to the 2010 MIS sampling in the archetype was measured in each simulation, generating for each archetype a series of *Pf*PR measurements for each larval habitat scale factor. The simulated *Pf*PR measurements were compared to measured *Pf*PR for the 2010 MIS using a beta binomial likelihood function and the scale factor resulting in the highest log-likelihood was chosen. Each LGA was assigned the seasonality profile and larval habitat scale factor of its archetype, upon which an LGA-specific set of interventions covering the period 2010–19 was imposed.

### Parameterizing historical interventions (2010–2019)

**Fig. 4:**
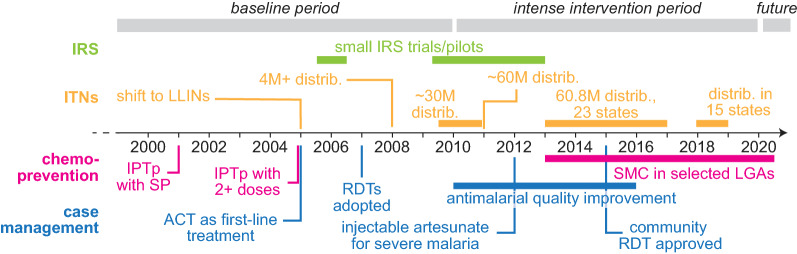
Timeline of interventions in Nigeria 2000–2020 used to inform model inputs

Historical interventions were parameterized for the *intense intervention period* from 2010 to 2019 when many interventions were scaled up in Nigeria (Fig. 4). Key historical intervention characteristics captured in the model were intervention type, timing, coverage, and effect size. Simulated intervention types were CM for uncomplicated and severe malaria, ITNs through household mass distribution and antenatal distributions, seasonal malaria chemoprevention (SMC), and intermittent preventive treatment in pregnancy (IPTp). Indoor residual spraying (IRS) was not included in the model since spraying activities were mostly trials or pilots covering a small subset of households [5, 48–50].

#### Case management (CM)

**Fig. 5:**
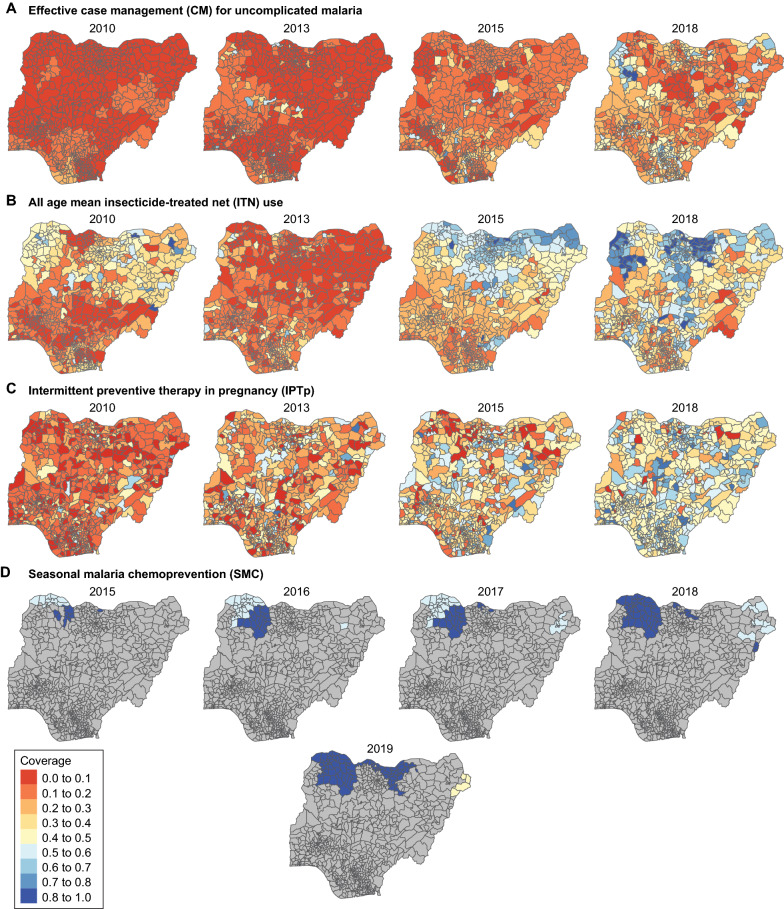
Intervention coverage levels used to parameterize the malaria transmission model from 2010–2019. **A** Effective CM rate for uncomplicated malaria. **B** ITN usage. **C** Coverage of one or more doses of IPTp. **D** SMC coverage per cycle, averaged across all four cycles. CM, ITN and IPTp were parameterized using data from the DHS while SMC was parametrized using programme data. The latest DHS survey was conducted in 2018

The 2010, 2013, 2015, and 2018 DHS/MIS were used to estimate time-varying effective CM coverage for uncomplicated malaria by LGA (Fig. 5A). CM coverage was calculated as the fraction of children under 5 with fever in the 2-week period prior to the survey treated with an artemisinin-based combination therapy (ACT). Since the DHS does not collect CM coverage data for adults, the same CM coverage was assumed for adults as for children based on a 2013 study where patent medicine vendors were roughly equally as likely to treat adults and children exhibiting malaria symptoms with an ACT [27]. CM coverage estimates at the LGA level were weighted using the cluster level weights, provided within the DHS dataset. Missing CM data per LGA were replaced with the archetype-level estimate, and CM during years between DHS/MIS surveys or after 2018 used values from the most recent survey. Effective CM coverage for severe malaria was held constant at 49% for all LGAs, based on reports that injectable artesunate is insufficiently prescribed [51], not readily available in health facilities [52], and may not be sufficient in the absence of additional supportive care [53]. For almost all modelled LGAs, CM for uncomplicated malaria was lower than for severe malaria with the exception of 33 LGAs in 2018. Treated malaria cases in the model received artemether-lumefantrine with an exponential treatment delay distribution averaging 3.3 days for uncomplicated cases and 2 days for severe cases.

#### Insecticide-treated nets (ITNs)

ITN coverages by age for simulated mass distributions were estimated from the 2010, 2013, 2015 and 2018 DHS/MIS surveys for the age groups 0–5, 6–9, 10–18, and > 18 years. ITN usage from the DHS/MIS is the fraction of individuals who slept under a treated bednet the night before the survey (Fig. 5B). Seasonal variation in ITN use was computed by aggregating DHS data between 2003 and 2018 and estimating monthly net use fractions. Among net users, a random 10% each night were assumed to not use their nets. ITN coverage from surveys was inflated using retention time estimates from MAP to obtain the initial ITN coverage at mass distribution, and net loss was also modelled according to half-life estimated by MAP [27]. The yearly mass distribution schedule at the LGA level was obtained from the NMEP and implemented in the simulation. However, the actual campaigns dates were assumed. LGA-level ITN coverage among pregnant women attending antenatal care in 2018 was calculated from program data shared by the NMEP and used within the simulation to distribute nets to newborns.

Nigeria’s mass campaigns have historically distributed pyrethroid ITNs. An ITN blocking rate of 0.53 was estimated from published experimental hut trials [54–56] and assumed to be uniform for these nets. ITN killing efficacy was estimated by analyzing the relationship between permethrin mortality and killing efficacy [57], which allowed parameterization by LGA and year with modelled maps of permethrin mortality for years 2005, 2010 and 2017 obtained from MAP [58] (see Additional file 1).

#### Intermittent preventive treatment in pregnancy (IPTp)

Dose-specific IPTp coverage in each LGA was estimated from self-reported receipt of one, two, or three or more doses of sulfadoxine-pyrimethamine (SP) in the DHS/MIS for years 2010, 2013, 2015, and 2018 (Fig. 5C). Coverage for years between surveys and up to 2020 was constructed using a monotone Hermite spline [59] (See Additional file 1).

Parasite prevalence outputs from the simulation were adjusted for the direct effect of IPTp among pregnant women, accounting for dose dependencies. Based on Menéndez et al*.* [60], one IPTp dose was estimated to provide complete protection against infection for 10 weeks, and each subsequent dose, up to three doses, added an additional 4 weeks of protection. Using these parameters, and assuming a 36-week pregnancy, the fraction of pregnancy that is unprotected for individuals who take one, two and three IPTp doses was estimated and used to compute adjusted prevalence estimates for pregnant women in LGA and for each simulation month. Final parasite prevalence estimates were calculated as the weighted average of the prevalence estimates in all groups, including pregnant and non-pregnant individuals (See Additional file 1).

#### Seasonal malaria chemoprevention (SMC)

Nigeria follows WHO recommendations to target SMC in seasonal areas where more than 60% of transmission occurs within 4 months and annual *PfPR* is greater than 5% [61]. Although the LGAs receiving SMC from 2013–2017 and the number of SMC doses distributed were known, reported coverage of at least one dose exceeded 100% and the lack of a reliable population denominator made it challenging to re-estimate coverage from dose data. Post-campaign surveys were instead used to parameterize SMC coverage [62, 63]. SMC coverage was assumed to be semi-correlated between rounds, as has been observed in post-SMC surveys where the fraction of children who receive all rounds of SMC is greater than would be expected from random selection per round [8, 62]. “High access” children in the model were assumed to be more likely to receive SMC doses than “low access” children. In the simulations, children were randomly assigned to access groups at birth.

For 2013–17, 50% of U5 children were assumed to be high access and the remaining 50% low access. To capture the national SMC coverage values of between 50 and 80% observed in post-campaign surveys [8] (Fig. 5D), the high access group received 80–100% SMC coverage per round and the low access group received 20–60% coverage per round. Coverages in a subset of LGAs in Zamfara state was set at 100% for the high access group and 60% in the low access group based on findings from a preliminary survey [64].

For 2018–19, survey data on overall state-level SMC coverage and fraction of children U5 in the high access group, defined as those who received all four SMC rounds [62, 63], for four states in 2018 and five states in 2019 was used to compute LGA-level coverage. However, some LGAs did not have low and high access children because survey data suggested that all children received the full course of SMC drugs. In those cases, children in the low access group had a coverage of 100%. The fraction in the high access group ranged from a low of 24% in Sokoto to a maximum of 53% in Zamfara. The fraction in the low access group was computed by subtracting the fraction in the high access group from the overall fraction of children (1 – fraction in high access group). LGA-level SMC coverage in the low access group did not vary within states and ranged from 20% to a high of 100%. For LGAs without survey data from 2018 to 19, SMC coverage from 2013 to 17 was used.

The pharmacokinetic parameters for the SMC drugs—sulfadoxine-pyrimethamine + amodiaquine—were obtained from the research literature [65–67] while pharmacodynamics properties were inferred through calibrations of simulated trials to clinical trial data from Ghana [68] and Tanzania [69]. Effect size of SMC on clinical incidence was validated using SMC rollout data from Mali [70]. All children were assumed to fully adhere to the 3-day treatment course.

### Parameterizing effect sizes for new interventions in future scenarios (2020–2030)

#### New net formulations

The NMEP considered pyrethroid piperonyl butoxide (PBO) and Interceptor® G2 (IG2, α-cypermethrin and chlorfenapyr) nets for future deployment. PBO effect sizes were assumed to increase with increasing susceptibility to pyrethroids [57] and were LGA-specific, but IG2 effect was modelled as uniformly high. See Supplement for details on parameterization of kill rates and blocking rates for new nets.

#### Intermittent preventive treatment in infants (IPTi)

IPTi (now known as PMC, perennial malaria chemoprevention), historically not implemented in Nigeria, was considered in 374 eligible LGAs as part of the national strategic planning exercise for 2021–2025. Eligibility was assessed by identifying LGAs not meeting WHO recommendations for SMC and with *Pf*PR > 10% in the absence of interventions [internal communication from WHO]. A modelled surface from MAP was used to assess *Pf*PR eligibility. IPTi effects were applied to simulation outputs rather than incorporated dynamically. Protective efficacy parameters for IPTi were extracted from a recent systematic review [71] and multiplied by LGA-specific coverage in each scenario to generate IPTi effects on prevalence, uncomplicated cases, severe cases, and deaths. LGA-specific expected coverage for IPTi was computed by taking the mean coverage of the first, second and third doses of pentavalent diphtheria, tetanus and pertussis vaccine from the 2018 DHS survey, in anticipation of IPTi administration coupled to the expanded program on immunization for children. IPTi coverage was further increased above the expected coverage as appropriate for each intervention scenario. See Additional file 1 for additional details.

#### Model validation

After setting model seasonality with the archetype aggregate of confirmed cases from the 2014–2018 RIA, and recapturing baseline transmission intensity in the pre-2010 model with archetype-level U5 P*f*PR from the 2010 DHS, simulations were run with historical interventions from the 2010–2018 period and the resulting U5 P*f*PR and all-age incidence outputs were validated by comparing with the DHS and health facility data at the state level. Model calibrations to capture transmission intensity were executed with only the 2010 DHS data to avoid confounding baseline transmission potential with intervention effects, as by 2015 interventions had been scaled up in many parts of Nigeria.

To validate the model, simulated U5 P*f*PR was aggregated to the state level and compared to state-level U5 P*f*PR data from the 2010 (calibration data), 2015 and 2018 DHS (out of sample data). The 2014–2018 RIA all-age monthly routine confirmed cases were aggregated to the state level and compared to the simulated malaria cases for individual states. Prevalence comparisons were done at the state level because the DHS is powered to be representative at the state level.

Prevalence comparisons were made with the Pearson correlation coefficient. For incidence, state-level comparisons were made between treated uncomplicated cases, including treated cases of non-malarial fevers in the simulation, and RIA health facility data (confirmed and suspected cases) using a cross-correlation function (CCF). The CCF at time lag zero is a measure of the contemporaneous correlation or the linear relationship between the two timeseries. Since the RIA data was constructed from a subset of facilities of unknown catchment size, incidence values were computed using the state population as denominator and rescaled to the simulation-observed range for visual comparison. Scale factors were computed by dividing the estimated LGA monthly incidence in the simulation by the corresponding LGA incidence estimated from the simulation. The median scale factor by state and year was used to adjust monthly incidence data from the RIA. Confidence intervals were computed from a non-parametric bootstrap with 10,000 replicates using the BCa method [72, 73].

#### LGA intervention allocation scenarios (2020–2030)

Predictions of the impact of intervention mixes on malaria prevalence, incidence, and mortality, within each of 774 LGAs, were simulated for four scenarios (Table 1) and for 5 stochastic realizations per scenario. The NMEP directed the type, coverage, and timing of interventions to be simulated. All scenarios deployed CM, IPTp, and ITNs in every LGA, but the distribution of SMC, IPTi, and ITN type varied by scenario, as did the effective coverage of each intervention. ITN distribution schedules were every three years from the last distribution year according to NMEP data. Here, “coverage” for ITNs refers to simulated usage. See Availability of data and materials for link to web app with scenario maps by LGA, intervention, and year. The scenarios were:

i. Business-as-usual (BAU, Scenario 1). SMC distribution and coverage of all interventions (CM, standard ITNs, SMC and IPTp) remained in the same LGAs and at the same coverage levels as in 2019. All LGAs received pyrethroid ITNs.
ii. National malaria strategic plan (NMSP) ramping up to 80% coverage (Scenario 2). PBO nets were distributed in all LGAs. All 404 LGAs that were eligible for SMC and 365 LGAs that were eligible for IPTi received SMC and IPTi, respectively. CM and IPTp were available in all LGAs. In Scenario 2, coverage of all interventions met or exceeded the program target of 80%. To simulate gradual intervention coverage improvement, coverage levels of CM and IPTp that were below the target of 80% in 2019 were increased over time to hit 80%, after which they were kept constant. Parameters from a beta regression model, used to estimate the average increase in CM coverage by archetype between 2013 and 2018, were scaled and added to the LGA-level 2019 coverage, until the 80% target was achieved. IPTp coverage increase was estimated with a linear regression model fitted to 2015–18 coverage data, except in LGAs where IPTp coverage had decreased in recent years, where IPTp coverage was held constant for 2018–30. Coverage of ITNs, SMC, and IPTi was set to 80% for LGAs where baseline coverage (or expected coverage for IPTi) was under 80%.
iii. Budget-prioritized plans submitted to the Global Fund (Scenarios 3, 4). Scenario 3 prioritized the implementation of SMC in 235 LGAs within the allocated budget, while scenario 4 included an additional 75 LGAs targeted for SMC in the priority above allocation request (PAAR) on top of the 235 LGAs in scenario 3. Other than SMC, interventions were the same in scenarios 3 and 4. CM and IPTp coverages were assumed to increase at historical rates without exceeding 80%. Rates of increase were parameterized using the same beta regression model parameters referenced in #ii above.. SMC and ITN coverages were simulated at 80%. Three net types were distributed in these scenarios: pyrethroid-only ITNs in 136 LGAs, PBO in 605 LGAs, and IG2 in 33 LGAs.

**Table 1:** Intervention scenarios chosen by the NMEP for simulation modelling

Scenario	Number of LGAs targeted for each intervention						
	CM	Pyr ITN	PBO ITN	IG2 ITN	IPTp	SMC	IPTi
1: Business-as-usual (BAU)	774	774			774	80	
2: NMSP ramping up to 80% coverage	774		774		774	404	365
3: Budget-prioritized plan with coverage increases at historical rate	774	136	605	33	774	235	
4: Budget-prioritized plan with coverage increases at historical rate and expanded SMC in the PAAR	774	136	605	33	774	310	

Numbers indicate number of LGAs receiving the intervention

*CM* case management, *Pyr ITN* Standard insecticide-treated nets/pyrethroid-only nets, *PBO* Pyrethroid + piperonyl butoxide nets, *IG2* (α-cypermethrin and chlorfenapyr) nets, *IPTp* intermittent treatment in pregnancy, *SMC* seasonal malaria chemotherapy, *IPTi* intermittent preventive treatment in infants, *PAAR* priority above allocation request

For each scenario, average trends in prevalence by microscopy, clinical incidence of uncomplicated malaria including both treated and untreated cases, and malaria-attributable mortality across all stochastic realizations were generated for U5 children and for all ages for years 2020–30. Case fatality rates were based on a review of published studies [74–77] and account for mortality due to severe malaria, maternal mortality from severe anemia in pregnancy, and mortality from malaria-attributable low birth weight (see Additional file 1). The 2020–30 projections for each scenario were compared against the simulated results for 2015 and against the 2020 BAU scenario to report projected relative difference in prevalence, uncomplicated malaria incidence, and malaria mortality among all ages and U5s for years 2025 and 2030.

## Results

To predict how each of the intervention scenarios would affect parasite prevalence, uncomplicated incidence, and malaria-attributable mortality between 2020 and 2030, a model was constructed for each LGA that captured observed seasonality and intensity of transmission, intervention history, and vector susceptibility to vector control. State- and national-level predictions were produced by aggregating LGA results.

### Model validation

**Fig. 6:**
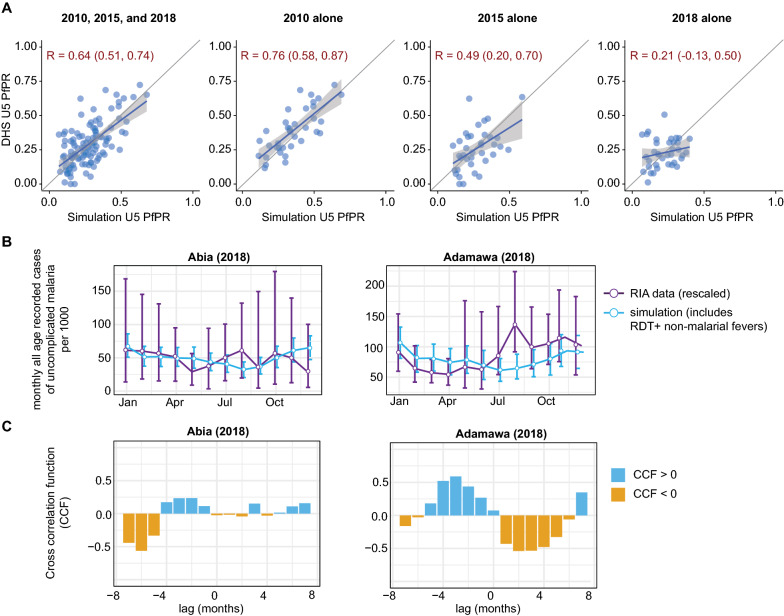
Validation. **A** Comparison of state-level U5 PfPR from DHS surveys with simulation values, matched by year and month. Pearson correlation coefficients with 95% confidence intervals are shown in red. **B** 2018 malaria seasonality in the RIA data (routine health facility data) and simulation for two states. Incidence values in the RIA data were scaled by the median relative difference between the simulation and RIA data by state. Purple vertical lines represent 95% confidence data for monthly values. Blue vertical lines are the simulation ranges computed from 5 stochastic realizations. **C** Comparison of RIA data and simulation seasonality trends with a cross-correlation function

Overall, the correlation between the observed and simulated prevalence and incidence data was weak. Although comparison of state-level U5 *Pf*PR from the 2010, 2015 and 2018 DHS to simulated measures from the same month and year of data collection indicated a strong positive correlation (R = 0.64, 95% CI, 0.51–0.74) (Fig. 6A), this was due to the 2010 data, which was used in fitting the simulated U5 *Pf*PR at the archetype level, which is the grouping of LGAs by epidemiological similarity, hence the higher magnitude of the Pearson correlation coefficient between 2010 values (R = 0.76, 95% CI, 0.58–0.87). The correlation between data and simulated values was substantially diminished in 2015 (R = 0.49, 95% CI 0.20–0.70) and 2018 (R = 0.21, 95% CI − 0.13–0.50).

Seasonality trends (Fig. 6B) and cross-correlation functions (Fig. 6C) of all-age monthly treated uncomplicated cases for Abia and Adamawa in 2018, states located in the South East and North East with differences in ecology, health-seeking and health system infrastructure [4, 78], showed a weak negative contemporaneous correlation between point estimates of observed and simulated cases in Abia (CCF = − 0.02) and a weak positive correlation in Adamawa (CCF = 0.06) at time lag zero. However, there was substantial uncertainty in the RIA data (Fig. 6B). Cross-correlations for all 37 states are shown in the Additional file 1.

### National-level predictions of scenario impact

**Fig. 7:**
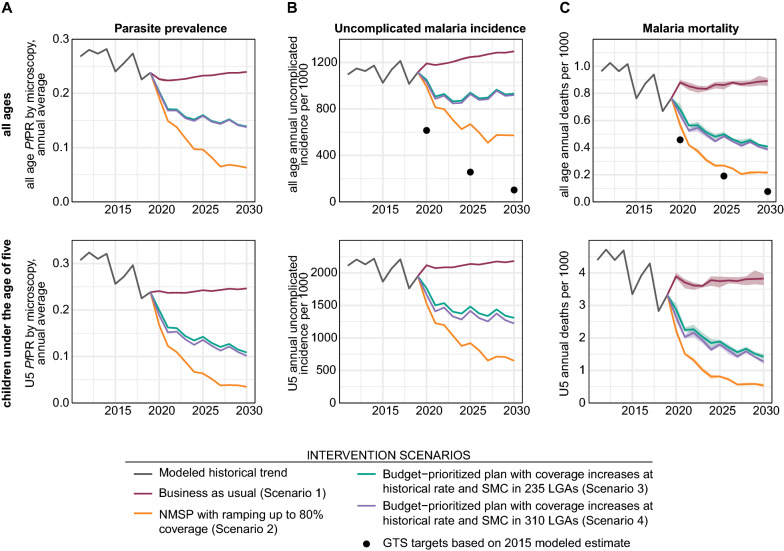
Impact predictions for 2020–2030 among individuals of all ages (top row) and among children under the age of five (bottom row). **A** Impact of interventions on *Pf*PR. **B** Impact of interventions on uncomplicated malaria incidence. **C** Impact of interventions on malaria mortality. Shaded areas represent impact prediction ranges based on five stochastic realizations

The NMSP at high effective coverage (Scenario 2 in Table 1) resulted in the greatest reductions in parasite prevalence, malaria incidence and mortality by 2030 (Fig. 7). Model predictions indicated that continued intervention deployment with a business-as-usual (BAU) strategy (Scenario 1) may lead to increases in all outcomes by 2030. The budget-prioritized plans in Scenarios 3 and 4 had an intermediate impact between Scenarios 1 and 2. Interactive visualizations of national, state and LGA-level predictions of scenario impact can be viewed and downloaded via a web app (see Availability of data and materials).

#### Parasite prevalence

Overall, small yearly increases in all-age *Pf*PR were observed in the BAU scenario (Scenario 1) after 2022 (Fig. 7A). National-level annual average all-age parasite prevalence was estimated at 0.24 for 2019 and predicted to decline by a relative 3% in 2025 and 0.1% by 2030 in Scenario 1.

Comparing the projected 2020 prevalence estimates in Scenario 1 to estimates in the same scenario and other scenarios in 2025 and 2030 (GTS target years), parasite prevalence among all ages and in U5s was projected to decline the most if the NMSP were implemented at high effective coverage (Scenario 2). Among U5 children, marginally greater reductions in prevalence were observed in Scenario 4 compared to Scenario 3 at national level (Table 2).

**Table 2:** Percent change in *Pf*PR in 2025 and 2030 relative to the BAU 2020 projected *Pf*PR

Scenario	All age *Pf*PR percent change in 2025 relative to BAU 2020 (%)	U5 *Pf*PR percent change in 2025 relative to BAU 2020 (%)	All age *Pf*PR percent change in 2030 relative to BAU 2020 (%)	U5 *Pf*PR percent change in 2030 relative to BAU 2020 (%)
1	2.8 (2.4, 3.1)	0.8 (0.2, 1.3)	5.8 (5.5, 6.2)	2.4 (2, 2.7)
2	− 57.4 (− 57, − 57.8)	− 73.6 (− 73.4, − 73.8)	− 72.1 (− 71.8, − 72.3)	− 85.6 (− 85.4, − 85.9)
3	− 29.3 (− 28.8, − 29.8)	− 40.8 (− 40.3, − 41.5)	− 38.6 (− 38.1, − 38.9)	− 54.9 (− 54.8, − 55.2)
4	− 30.0 (− 29.6, − 30.2)	− 43.6 (− 43.2, − 44.2)	− 39.1 (− 38.7, − 39.4)	− 57.9 (− 57.7, − 58.1)

Projected ranges based on five stochastic realizations

#### Uncomplicated malaria incidence

Total modelled uncomplicated malaria incidence was the sum of simulated treated and untreated uncomplicated cases. The GTS reduction targets for global malaria case incidence are a minimum of a 40%, 75% and 90% declines in 2020, 2025 and 2030 relative to a 2015 baseline [2]. Overall, simulation results suggested that none of the scenarios were likely to meet these targets (Fig. 7B). Comparison of projected all-age incidence for the GTS target years with the estimated 2015 baseline of 1025 cases per 1000 showed that the greatest reductions may occur in Scenario 2, where incidence decreased by 44% in 2030.

All-age incidence in Scenario 1 increased by 5% and 9% in 2025 and 2030, respectively, relative to its 2020 estimated value (Table 3). Relative to Scenario 1 in 2020, Scenario 2 delivered the greatest reductions in all-age and U5 incidence in 2025 and 2030. Very small relative differences in incidence were observed between Scenario 3 and 4 among all ages and among U5 in 2025 and 2030.

**Table 3:** Percent change in incidence in 2025 and 2030 relative to the BAU 2020 projected incidence

Scenario	All age incidence percent change in 2025 relative to BAU 2020 (%)	U5 incidence percent change in 2025 relative to BAU 2020 (%)	All age incidence percent change in 2030 relative to BAU 2020 (%)	U5 incidence percent change in 2030 relative to BAU 2020 (%)
1	4.7 (3.8, 5.3)	0.9 (0.3, 1.5)	8.7 (8.4, 9.1)	3.0 (2.6, 3.3)
2	− 43.9 (− 43.3, − 44.3)	− 56.6 (− 56.2, − 56.8)	− 51.9 (− 51.4, − 52.4)	− 69.3 (− 69, − 69.6)
3	− 21.1 (− 20.6, − 21.8)	− 30 (− 29.4, − 30.5)	− 21.8 (− 21.3, − 22.3)	− 38.2 (− 38, − 38.4)
4	− 22.1 (− 21.6, − 22.6)	− 33.1 (− 32.7, − 33.4)	− 22.8 (− 22.2, − 23.5)	− 42.2 (− 41.8, − 42.6)

Projected ranges based on five stochastic realizations

#### Malaria mortality

The GTS has reduction targets of a minimum of 40%, 75% and 90% decline in mortality in 2020, 2025 and 2030 relative to a 2015 baseline [2]. Scenario 2 was projected to deliver the greatest reductions in all-age mortality relative to the 2015 baseline and would likely meet the GTS 2025 targets, as mortality declines of 65% were predicted for that year. Projected all-age mortality rates were highest in Scenario 1, where mortality rates increased by 15% in 2020, 13% in 2025, and 17% in 2030 compared to 2015 (Fig. 7C).

Relative to the projected mortality rate in Scenario 1 in 2020, mortality rates in 2025 and 2030 declined the most in Scenario 2 for all ages and U5 (Table 4). Mortality rates declined by a smaller but still substantial amount in Scenarios 3 and 4.

**Table 4:** Percent change in mortality in 2025 and 2030 relative to the BAU 2020 projected incidence

Scenario	All age mortality percent change in 2025 relative to BAU 2020 (%)	U5 mortality percent change in 2025 relative to BAU 2020 (%)	All age mortality percent change in 2030 relative to BAU 2020 (%)	U5 mortality percent change in 2030 relative to BAU 2020 (%)
1	− 2.2 (0.5, − 5.9)	− 3.8 (0.3, − 9.1)	1.2 (4.1, − 4.6)	− 1.7 (2.4, − 9.5)
2	− 69.6 (− 68.8, − 70.3)	− 79.0 (− 78.2, − 79.9)	− 75.6 (− 74.1, − 76.6)	− 86.1 (− 84.6, − 87.5)
3	− 43.4 (− 40.6, − 45.7)	− 51.4 (− 50.3, − 54.2)	− 53.8 (− 52.6, − 54.3)	− 63.4 (− 61.1, − 65)
4	− 45.1 (− 41.9, − 46.0)	− 53.8 (− 51.9, − 55.1)	− 56.1 (− 54.1, − 57)	− 67.2 (− 65.3, − 68.9)

Projected ranges based on five stochastic realizations

### Highlights from the state-level predictions

Scenario 2, which was projected to result in the greatest reductions in malaria outcomes for all years at the national level, also performed best in 2025 for all indicators in 36 of the 37 states. Anambra state in the South-East of Nigeria was the only state where Scenario 3 or 4 (equivalent scenarios in this state since SMC was not part of the intervention mix) performed best in 2025. Outcomes in 2030 were similar. Scenario 2 performed best for all indicators in 34 states. In Anambra and Kwara states, Scenario 3 and Scenario 2, respectively, was the best performing scenario for five of the six indicators whereas, Scenario 4 was the optimal scenario for four of the six indicators in Kebbi state.

### Impact of SMC expansion in the budget-prioritized scenarios

To highlight the impact of expansion in the budget-prioritized scenarios, U5 incidence and mortality in the 310 SMC-eligible LGAs in Scenario 3 (235/310 LGAs received SMC) and Scenario 4 (310/310 LGAs received SMC) were compared to Scenario 1 (80/310 LGAs received SMC).

Whereas both Scenarios 3 and 4 were projected to show greater improvements in U5 malaria burden than Scenario 1, Scenario 4 outperformed Scenario 3 in projected declines in indicators in 2025 and 2030 relative to 2020 BAU (Table 5). Relative comparison of incidence and mortality outcomes in 2030 between Scenario 3 and 4 suggested that Scenario 4 may result in an additional 9% and 8% decline in U5 incidence and mortality in SMC-eligible areas, respectively.

**Table 5:** Percent change in indicators in 2025 and 2030 relative to the BAU 2020 scenario in 310 SMC-eligible areas

Scenario	U5 incidence percent change in 2025 relative to BAU 2020 (%)	U5 mortality percent change in 2025 relative to BAU 2020 (%)	U5 incidence percent change in 2030 relative to BAU in 2020 (%)	U5 mortality percent change in 2030 relative to BAU 2020 (%)
1	− 4.4 (− 3.8, − 5.3)	− 12.7 (− 7.1, − 18.5)	0.5 (1.1, − 0.2)	− 6.5 (1.9, − 12.7)
3	− 35.7 (− 34.8, − 36.3)	− 54.3 (− 51.7, − 57.7)	− 34.7 (− 34.1, − 35.5)	− 61.2 (− 58.3, − 63.4)
4	− 42.4 (− 42.2, − 42.7)	− 59 (− 55.6, − 63.2)	− 43.4 (− 42.9, − 43.7)	− 68.9 (− 67.1, − 70.8)

## Discussion

In partnership with WHO, Nigeria developed subnationally-tailored intervention plans for their NMSP and Global Fund requests finalized in the fourth quarter of 2020. A suite of 774 LGA-level models of malaria transmission was developed to predict the impact of the NMSP and budget-prioritized intervention packages on malaria prevalence, incidence, and mortality. This is the first application of mathematical models to inform subnational strategy in Nigeria during NMSP development. With the support of Scenario 3 and 4 predictions, Nigeria obtained funds for the expansion of SMC from 80 to 310 LGAs, covering over 36 million children in all four SMC rounds in 2021, and for the distribution of PBO nets to cover over 160 million people between 2021 and 2025 [1] (email communication from WHO).

Scenario 2 was projected to likely meet the 2025 GTS mortality target, but Scenarios 3 and 4 were not projected to meet any of the GTS 2025 and 2030 targets. The modelled ITNs had only limited impact due to insecticide resistance, outdoor biting, imperfect individual usage, and short retention time. There is considerable uncertainty regarding each of these quantities, and the actual impact of ITN distribution could well be greater than model predictions. Additional strategies, such as community engagement to increase intervention acceptability and adherence, improved access to drug-based prevention and treatment, or different approaches to vector control are needed in areas with high vectorial capacity or substantial biting when people are unprotected by ITNs. The current mix and coverage of programmatic interventions in malaria-endemic countries may not interrupt *P. falciparum* transmission, necessitating new strategies [13, 79].

Major limitations to this project fall in a few broad categories: model structure, uncertain intervention effect sizes, insufficient LGA-level data, and the absence of meaningful uncertainty metrics. The impact of these limitations is evident in the poor fit between model predictions and DHS data of U5 *Pf*PR after 2010, as well as the discrepancy between the malaria seasonality from routine data and predicted seasonality at the state level. Several simplifying assumptions were made to increase the tractability of model development. Modeled agents were assumed to mix within the LGAs, but not between LGAs, and a uniform importation rate was assumed for all LGAs and all years of simulation. These assumptions may hold for higher-transmission LGAs but likely do not for lower-transmission LGAs [80]. The model did not account for long-term trends in economic development, urbanization, housing improvements, or environmental management, which may well reduce malaria risk for many Nigerians over the next decade. The modeling framework did not include effects of climate change, year-to-year variation in climate, intervention-delivery dynamics, or service disruption due to COVID-19 or insecurity. With regards to children under the age of five, the timing and duration of infection was assumed to vary minimally, which affects the predicted effects of SMC for this group. Model predictions of malaria deaths depend on the treatment rate of severe malaria and mortality rates of treated and untreated severe cases, all of which were based on very limited or scattered data [74, 81–83]. Finally, emerging data on the infectivity of chronic asymptomatic infections suggests that the model’s case management effect size may be too large [84].

Estimates of the efficacy of SMC and ITNs were based on data from clinical trial sites rather than field data from Nigeria. Pyrethroid and PBO ITN effect sizes depended on pyrethroid mortality rates from a geospatial model rather than from local field data, and experimental hut data underlying the relationship between mortality rate and ITN effect size was extremely noisy. Model parameters were also limited by insufficient literature on vector bionomics, spatial variation in species composition, and the seasonality of individual species in Nigeria and across the region. These key entomological parameters also influence ITN effect size.

The estimates of effective CM coverage from the DHS/MIS may not adequately reflect ground truth. The cross-sectional nature of the survey only captures ACT use among those with fever within the 2-week period that the survey is conducted. Survey reports of fevers may include non-malarial fevers for which ACT use is not relevant. State-level estimates from the 2018 DHS, Nigeria’s most recent survey at the time of this study, suggest very low CM rates, in contrast with the view of the NMEP after consultation, and with 2015 ACTwatch data showing good availability of ACT [85, 86]. Very low effective CM coverage in the model will skew prevalence and mortality levels higher and increase the potential impact of raising CM coverage to target levels.

The lack of LGA-level prevalence measurements, reliable incidence data, and intervention coverage data limited this study’s ability to develop accurate LGA-level models and validate them. Because it was impossible to estimate baseline transmission intensity at the LGA level, geospatial model estimates were used to group LGAs into epidemiological archetypes. The use of routine incidence data to depict seasonality patterns at the LGA and archetype level was challenged by data quality and representability, likely affected by data entry and reporting errors, preferential care-seeking in the private sector, or facility accessibility issues [87]. The probability sampling methods used by the DHS makes it an improved data source for estimating transmission intensity over routine data but since it is insufficiently representative at the LGA-level [86], archetype-level estimates was a reasonable alternative. However, archetype-level calibrations would not fully capture between-LGA variations in transmission indicators. The lack of reliable LGA-level data on effective CM coverage, ITN usage, and SMC coverage meant that heterogeneous coverage within states or archetypes often could not be captured, or coverage may have been mischaracterized by using an LGA-level estimate from DHS when DHS is not powered at the LGA level.

Due to the need to develop the technical framework from scratch and meet the NMEP’s deadlines, it was not feasible to quantify the impact of uncertainty in model parameters or model structure on scenario outcomes. Predicted estimates have high uncertainty and should not be taken as exact future prediction. Relative estimates are more robust and appropriate to consider for decision-making. Future iterations of NMSP modelling will build on the work presented here and incorporate uncertainty propagation and sensitivity analyses. Irrespective of data limitations, national malaria programs need to make intervention allocation decisions. These models combine the best available information into a testable framework where assumptions could be examined and corrected. Despite its limitations, this model was able to quantitatively assess the impact of proposed plans relative to GTS targets, allow relative comparison of candidate plans, provide evidence to advocate for additional resources, and set feasible expectations of outcomes. Overall, the model predicts that improving coverage of existing interventions would have considerable impact on malaria morbidity and mortality. The readily available calibrated model can be used to test new scenarios and extended to answer additional questions to inform resource reallocation decisions during annual progress reviews and national strategic planning cycles. The modelling framework has been adapted for use in other malaria-endemic countries.

By being transparent about the model’s shortcomings, this project highlights the need for increased investments in the collection of subnational surveillance data and in research to enhance understanding of spatial variance in malaria transmission, intervention coverages and effects, and entomology. High-quality data is required for evidence-driven and appropriately targeted national plans, and modelling cannot compensate for insufficient data. Subnational monitoring and evaluation of burden and intervention impact are in process for additional countries in the next cycles of planning, and data gathered during these processes will also be crucial for validating and improving models. High-quality models could be used to attribute intervention impact during progress reviews, quantify the effectiveness of new interventions, and potentially contribute to program evaluation. Quality standards, rigorous model validation, and stakeholder buy-in are necessary before models should be applied for evaluation. Even if data limitations are overcome, the utility of fine-scale malaria transmission models in programmatic decision-making will not be fully realized if there is no consensus on requirements for a high-quality and appropriate model, or if models are too rigid to adapt for new questions. Multiple models generating qualitatively different predictions for the same question will diminish the credibility of modelers and programmatic trust in model outputs. Sustaining and expanding the use of models to inform decision-making in malaria programmes will require flexible and user-friendly models capable of answering a broad range of questions and building the capacity of local staff and researchers to develop models for their specific questions.

## Conclusions

Mathematical modelling provides a framework for quantitative assessment of the impact of future intervention scenarios. As part of the HBHI response implemented in Nigeria, LGA-specific malaria transmission models were developed and used by the Nigeria NMEP to inform decisions on the optimal and financially feasible intervention mix from four alternatives. Investment into subnational data on malaria burden and intervention effects is urgently needed to allow data-driven impact modelling at a finer scale and improve decision-making on optimal malaria intervention mixes at the subnational level.

## Supplementary Information

**Additional file 1: Figure S1.** Assignment of 774 LGAs in Nigeria into 22 epidemiological archetypes. **Figure S2.** Simulated seasonality of clinical malaria by archetype compared with the Rapid Impact Assessment health facility data for years 2014 – 2018. Thin red lines show 50 stochastic realizations and solid red dots and line show the mean over the realizations. **Figure S3.** a: Case management among children under the age, insecticide treated nets use and PfPR among children under the age of five years in 2010 by LGA. b: Two plots are shown for each archetype with archetype names at the top of each plot. The left plot is the larval habitat multiplierand likelihood evaluation against archetype U5 PfPR 2010 MIS. The red dot is maximum likelihood estimate of LHM. The right plot is the simulated U5 PfPR within each archetype compared with monthly U5 PfPR from the 2010 MIS. The thick red line indicates the best match while thin red lines show PfPR under other larval habitat scale factors. Each line is the mean of 10 stochastic realizations. 12 out of the 22 archetypes are shown here, remainder are shown in Fig S3b. **Figure S4.** ITN coverage among pregnant women attending ANC in 2018. **Figure S5.** Estimated ITN kill rate for a 12% reduction in annual malaria incidence among children under the age of five years. **Figure S6.** The relationship between permethrin bioassay mortality and ITN killing rate in the Churcher et al. model and EMOD. Scale factors was calculated by dividing the EMOD kill rateby the kill rate from the Churcher et al. model. **Figure S7.** Fitted splines showing estimated IPTp coverage through time for a random subset of LGAs. Points show DHS/MIS data and lines show the fitted splines, with each color indicating a different LGA. **Figure S8.** Fraction of IPTp-receiving individuals who reported receiving one, two, or three or more doses in each DHSor MIS. **Figure S9.** LGAs designated as eligible to receive IPTi. **Figure S10.** Mean DTP1-3 vaccine coverage per LGA in the 2018 DHS survey. **Figure S11.** Archetype level scatterplot highlighting beta-regression model equations used to compute predicted average yearly change CM coverages per archetype. Annual case management is the proportion of children with fever in the 2 weeks period prior to the survey that received an ACT. **Figure S12.** Malaria seasonality in routine health facility data and simulation for 37 Nigerian states in 2014. Incidence values in the health facility data were scaled by the median relative difference between the simulation and RIA data by state. Vertical purple horizontal lines are 95% confidence intervals for the RIA data. Vertical blue lines are the ranges of the simulations from 5 seed runs. **Figure S13.** Comparison of DHIS2 and simulation seasonality trends in 2014 with a cross-correlation function. CCF at the time lag zero is a measure of the contemporaneous correlation or the linear relationship between the two time series. **Figure S14.** Malaria seasonality in routine health facility data and simulation for 37 Nigerian states in 2015. Incidence values in the health facility data were scaled by the median relative difference between the simulation and RIA data by state. Vertical purple horizontal lines are 95% confidence intervals for the RIA data. Vertical blue lines are the ranges of the simulations from 5 seed runs. **Figure S15.** Comparison of DHIS2 and simulation seasonality trends in 2015 with a cross-correlation function. CCF at the time lag zero is a measure of the contemporaneous correlation or the linear relationship between the two time series. **Figure S16.** Malaria seasonality in routine health facility data and simulation for 37 Nigerian states in 2016. Incidence values in the health facility data were scaled by the median relative difference between the simulation and RIA data by state. Vertical purple horizontal lines are 95% confidence intervals for the RIA data. Vertical blue lines are the ranges of the simulations from 5 seed runs. **Figure S17.** Comparison of DHIS2 and simulation seasonality trends in 2016 with a cross-correlation function. CCF at the time lag zero is a measure of the contemporaneous correlation or the linear relationship between the two time series. **Figure S18.** Malaria seasonality in routine health facility data and simulation for 37 Nigerian states in 2017. Incidence values in the health facility data were scaled by the median relative difference between the simulation and RIA data by state. Vertical purple horizontal lines are 95% confidence intervals for the RIA data. Vertical blue lines are the ranges of the simulations from 5 seed runs. **Figure S19.** Comparison of DHIS2 and simulation seasonality trends in 2017 with a cross-correlation function. CCF at the time lag zero is a measure of the contemporaneous correlation or the linear relationship between the two time series. **Figure S20.** Malaria seasonality in routine health facility data and simulation for 37 Nigerian states in 2018. Incidence values in the health facility data were scaled by the median relative difference between the simulation and RIA data by state. Vertical purple horizontal lines are 95% confidence intervals for the RIA data. Vertical blue lines are the ranges of the simulations from 5 seed runs. **Figure S21.** Comparison of DHIS2 and simulation seasonality trends in 2018 with a cross-correlation function. CCF at the time lag zero is a measure of the contemporaneous correlation or the linear relationship between the two time series.

## Data Availability

The RIA dataset and LGA-level population estimates is available on request from the Nigerian NMEP. Data from the Demographic and Health Surveys and Malaria Indicator Surveys can be obtained from https://dhsprogram.com/. Model inputs and scripts can be accessed from https://github.com/numalariamodeling/hbhi-nigeria-publication-2021. A Shiny app for exploring detailed model results is available at https://ifeomaozo.shinyapps.io/hbhi-nigeria/_w_40c53993/ (Note: username: fanny; password: azerty).

## Abbreviations

ACT: Artemisinin-based combination therapy
BAU: Business-as-usual
CM: Case management
CCF: Cross correlation function
DHS: Demographic and Health Survey
EMOD: Epidemiological Modeling software
GTS: Global technical strategy
HBHI: High burden to high impact
IRS: Indoor residual spraying
ITN: Insecticide-treated net
IPTi: Intermittent preventive treatment in infants
IPTp: Intermittent preventive treatment in pregnancy
LGAs: Local government areas
MAP: Malaria Atlas Project
MIS: Malaria Indicator Survey
NMSP: National Malaria Strategic Plan
NMEP: Nigeria National Malaria Elimination Programme
PAAR: Prioritized above funding request
PBO: Piperonyl Butoxide
*Pf*PR: *Plasmodium falciparum* Parasite rate (*Pf*PR)
RDT: Rapid Diagnostic Test
RIA: Rapid Impact Assessment
SMC: Seasonal Malaria Chemoprevention
TSI: Temperature Suitability Index
WHO: World Health Organization
