# Supplementary material for: Application of mathematical modelling to inform national malaria intervention planning in Nigeria
Source: Malar J. 2023 Apr 26;22:137. doi: 10.1186/s12936-023-04563-w (PMC10130303; doi:10.1186/s12936-023-04563-w)
Supplement: Supplementary file 1 — Additional file 1: Figure S1. Assignment of 774 LGAs in Nigeria into 22 epidemiological archetypes. Figure S2. Simulated seasonality of clinical malaria by archetype compared with the Rapid Impact Assessment health facility data for years 2014 – 2018. Thin red lines show 50 stochastic realizations and solid red dots and line show the mean over the realizations. Figure S3. a: Case management among children under the age, insecticide treated nets use and PfPR among children under the age of five years in 2010 by LGA. b: Two plots are shown for each archetype with archetype names at the top of each plot. The left plot is the larval habitat multiplierand likelihood evaluation against archetype U5 PfPR 2010 MIS. The red dot is maximum likelihood estimate of LHM. The right plot is the simulated U5 PfPR within each archetype compared with monthly U5 PfPR from the 2010 MIS. The thick red line indicates the best match while thin red lines show PfPR under other larval habitat scale factors. Each line is the mean of 10 stochastic realizations. 12 out of the 22 archetypes are shown here, remainder are shown in Fig S3b. Figure S4. ITN coverage among pregnant women attending ANC in 2018. Figure S5. Estimated ITN kill rate for a 12% reduction in annual malaria incidence among children under the age of five years. Figure S6. The relationship between permethrin bioassay mortality and ITN killing rate in the Churcher et al. model and EMOD. Scale factors was calculated by dividing the EMOD kill rateby the kill rate from the Churcher et al. model. Figure S7. Fitted splines showing estimated IPTp coverage through time for a random subset of LGAs. Points show DHS/MIS data and lines show the fitted splines, with each color indicating a different LGA. Figure S8. Fraction of IPTp-receiving individuals who reported receiving one, two, or three or more doses in each DHSor MIS. Figure S9. LGAs designated as eligible to receive IPTi. Figure S10. Mean DTP1-3 vaccine coverage per LGA in the 2018 [file 12936_2023_4563_MOESM1_ESM.docx]

**Additional material for Ozodiegwu et al. “Application of mathematical modeling to inform national malaria intervention planning in Nigeria”**

Contents

[Clustering LGAs into epidemiological archetypes 2](#_Toc77757573)

[Setting baseline transmission intensity by archetype 5](#_Toc77757574)

[Parameterizing intervention coverage 6](#_Toc77757575)

[Parametrizing the ITN model 7](#_Toc77757576)

[ITN blocking rate 8](#_Toc77757577)

[ITN killing rate 8](#_Toc77757578)

[*i.* *Permethrin (pyrethroid) nets* 8](#_Toc77757579)

[*ii.* *Pyrethroid piperonyl butoxide (PBO) nets* 10](#_Toc77757580)

[*iii.* *IG2 nets* 11](#_Toc77757581)

[Intermittent preventive treatment in pregnant women (IPTp) 11](#_Toc77757582)

[IPTp coverage 2010-2020 11](#_Toc77757583)

[Estimating the fraction of individuals that received different IPTp doses 11](#_Toc77757584)

[Adjusting PfPR to account for the impact of IPTp 12](#_Toc77757585)

[Intermittent Preventive Treatment in Infants (IPTi) 13](#_Toc77757586)

[Estimating likely IPTi coverage through historical vaccine coverage 13](#_Toc77757587)

[Adjusting prevalence, incidence and mortality for the impact of IPTi 14](#_Toc77757588)

[*i.* *Malaria infections, cases and deaths averted in infants due to IPTi* 14](#_Toc77757589)

[ii. *Malaria infections, cases and deaths averted in U5 and total population* 15](#_Toc77757590)

[*iii.* *Use of relative reductions per age group as scaling factors for IPTi effectiveness* 15](#_Toc77757591)

[Estimating malaria mortality 15](#_Toc77757592)

[Mortality from treated and untreated severe malaria 15](#_Toc77757593)

[Mortality from severe maternal anemia attributable to malaria in pregnancy 16](#_Toc77757594)

[Mortality from low birth weight attributable to malaria 17](#_Toc77757595)

[Intervention scenarios and predictions 18](#_Toc77757596)

[Case management (CM) coverage in intervention scenarios 18](#_Toc77757597)

[Validation 19](#_Toc77757598)

[Incidence comparison plots and cross-correlation plots by state 21](#_Toc77757599)

Modeling approach

1. Local Government Areas (LGAs) were grouped by shared seasonality and baseline transmission intensity to generate epidemiological archetypes
2. Monthly larval habitat availability in each of the epidemiological archetypes was calibrated to match the seasonality of treated cases within select health facilities.
3. The archetype models in b) were each calibrated in a second step to match observed transmission intensity under observed 2010 insecticide treated net (ITN) coverage. The baseline transmission intensity that best matched the 2010 *Plasmodium* *falciparum* parasite rate (*PfPR*) among children under the age of five from the Demographic and Health Surveys (DHS) was selected for each archetype, given its 2010 ITN coverage.
4. A post-2010 intervention history, including coverage of case management, ITNs, seasonal malaria chemoprevention (SMC), and intermittent preventive treatment in pregnancy (IPTp), was reconstructed for each LGA using data from the DHS and national sources.
5. With the model selected in c), each LGA was simulated through the end of 2019 in the presence of its intervention history, validating simulated *PfPR* against data from the DHS from 2014 and 2018 when possible.
6. Each LGA was simulated forward from 2020 – 2030 under various possible intervention scenarios. Model predictions of reductions in prevalence, cases, and deaths were compared for each scenario.

# Clustering LGAs into epidemiological archetypes

The CLARA algorithm [1,2] was implemented using the R package “cluster: Cluster Analysis Basics and Extensions” [3] to generate clusters of LGAs that shared similar climatic, vector, transmission and intervention features. To adjust the relative importance of the variables used to form these clusters, each category of input variables (e.g., rainfall, relative vector abundance) was assigned a weight (Table S1). Raster inputs were rescaled so that the standard deviation of all pixel values was equal to the category weight divided by the number of inputs in that category. The mean value of all pixels within the borders of an LGA (represented as *m_v,l_* for variable *v* and LGA *l*) was used for clustering.

Sixty clusters were generated by this process. The number of clusters was determined through an iterative process. We visually compared the input versus cluster-assigned values for all of the input variables (monthly rainfall, PfPR, etc.). Using too few clusters resulted in our not being able to capture the salient patterns in the input variables, while too many clusters would have been unfeasible to calibrate. Twenty clusters was determined to result in satisfactory pattern recapture. Since LGAs in clusters with small MIS sample sizes would need to be reassigned (process described below), we selected 60 as the number of clusters to be generated through CLARA. This resulted in 22 final archetypes that did a reasonable job at recapturing the patterns from the input parameters.

Because we relied on the 2010 MIS parasite prevalence survey results to calibrate each archetype’s transmission intensity, LGAs in archetypes that contained fewer than fifty surveyed children were reassigned to the next-best archetype. The next-best archetype for LGA *l* was selected as the archetype that that minimized:

$$\sum_{v \in input variables} \left( m_{v,l}-m_{v,r} \right)^{2}$$

where *r* is the representative LGA of the archetype (as identified by the CLARA algorithm). The resulting set of twenty-two archetypes is shown in Figure S1.

Table S1. Input variables and weights used to cluster LGAs into epidemiological archetypes.

| **Category** | **Category weight** | **Description** | **Number of input rasters** | **Weight for each raster** | **Reference** |
| --- | --- | --- | --- | --- | --- |
| Monthly rainfall | 3 | Total rainfall in each month | 12 | 0.25 | [4] |
| Monthly temperature suitability index | 2 | Average temperature suitability index in each month | 12 | 0.167 | [5] |
| Relative vector abundance | 2 | Relative abundance of three dominant vector species | 3 | 0.667 | [6] |
| Annual PfPR | 3 | MAP PfPR estimates from 2000, 2004, 2006, 2008, and 2010 | 5 | 0.6 | [7,8] |
| ITN use | 1 | MAP ITN estimates for 2008, 2009, and 2010 | 3 | 0.333 | [9] |


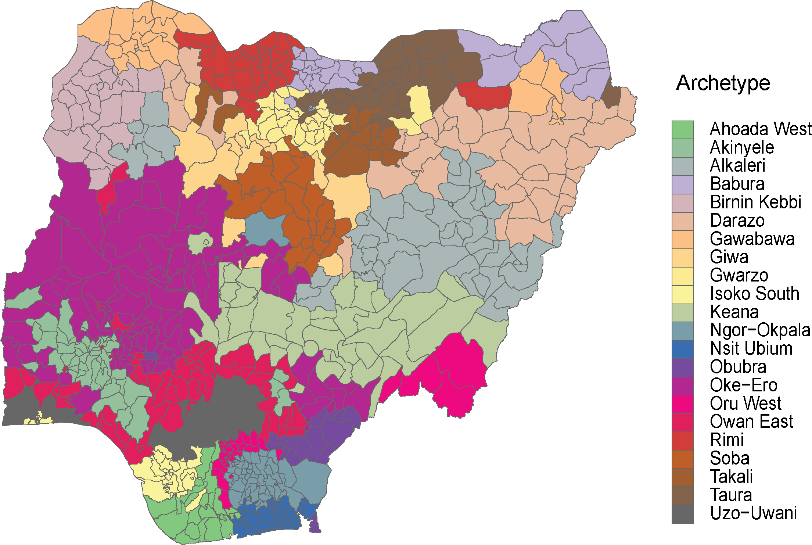


Figure S1: Assignment of 774 LGAs in Nigeria into 22 epidemiological archetypes.

Setting archetype seasonality


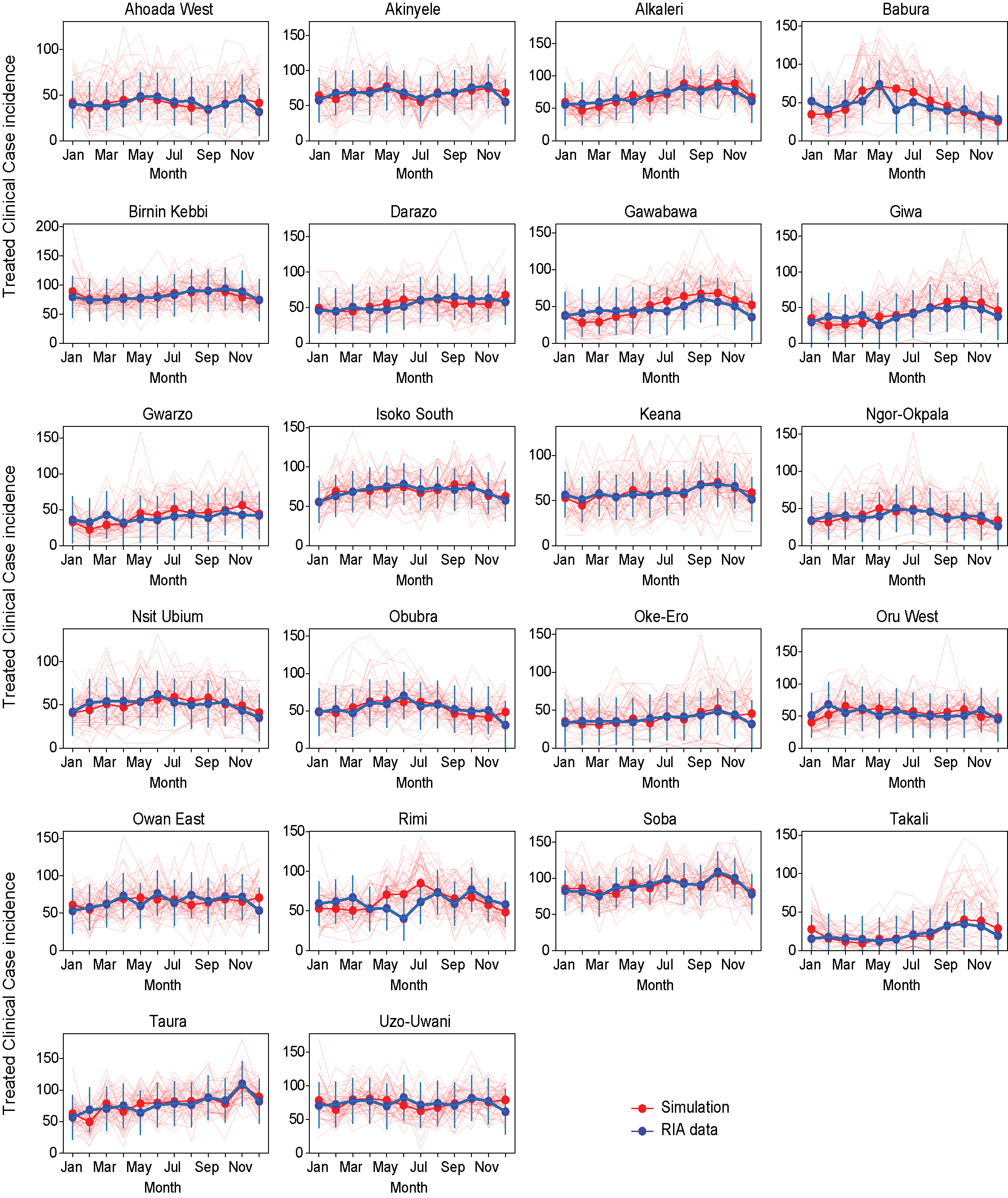


Figure S2: Simulated seasonality of clinical malaria by archetype compared with the Rapid Impact Assessment health facility data for years 2014 – 2018. Thin red lines show 50 stochastic realizations and solid red dots and line show the mean over the realizations.

# Setting baseline transmission intensity by archetype

Each larval habitat scaling factor on each epidemiological archetype was simulated for 50 years up to January 1, 2010 to capture population immunity. Because DHS is nor powered to produce representative estimates of case management, insecticide treated nets and prevalence of malaria among children under the age of five years at the LGA level (Figure 3a), archetype level estimates was used in the baseline calibrations. Case management coverage from the 2010 DHS was applied beginning in 2005. Simulated PfPR shown in Figure S3b correspond to the 2010 calendar year.


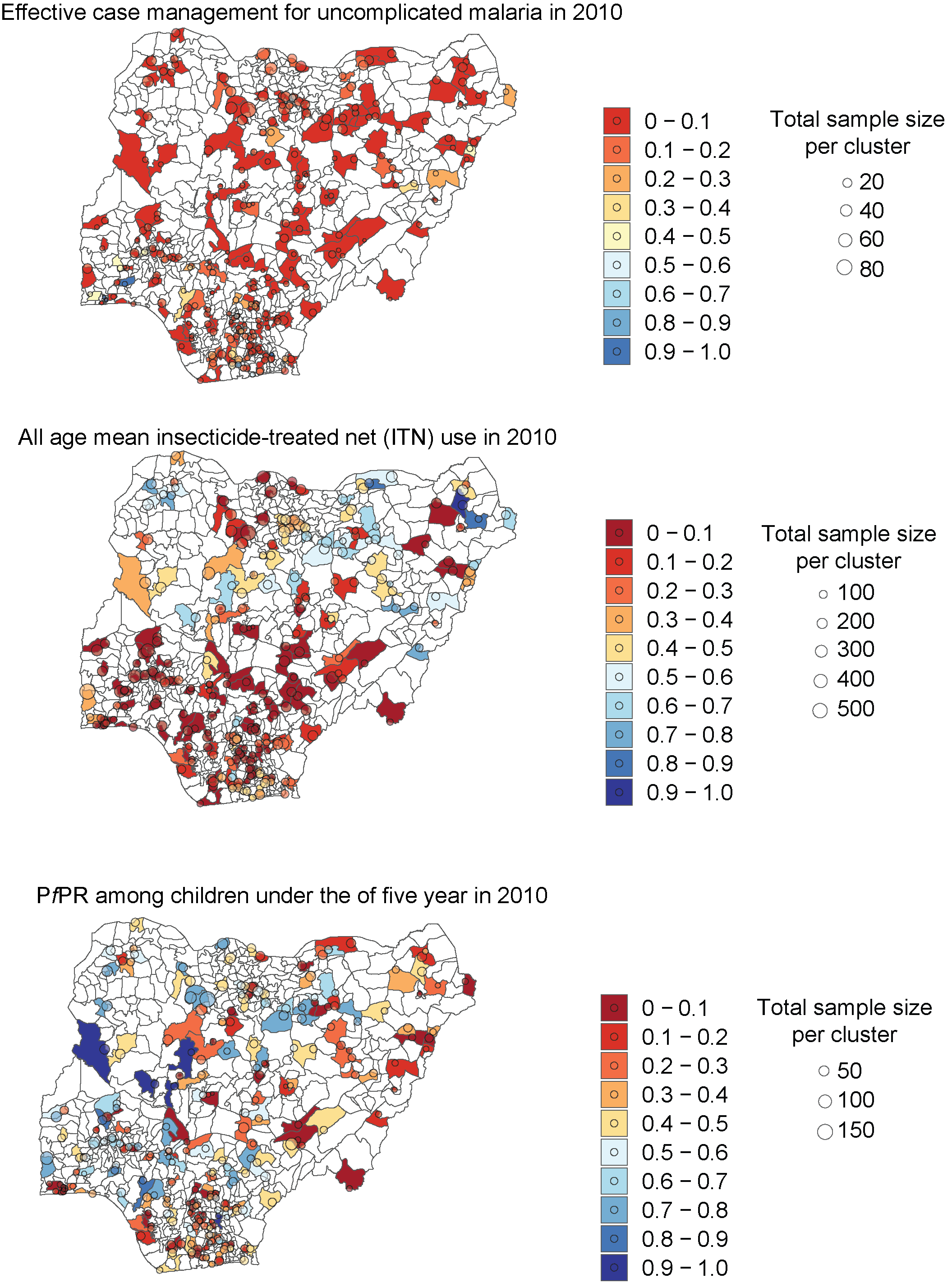


Figure S3a: Case management among children under the age, insecticide treated nets use and P*f*PR among children under the age of five years in 2010 by LGA


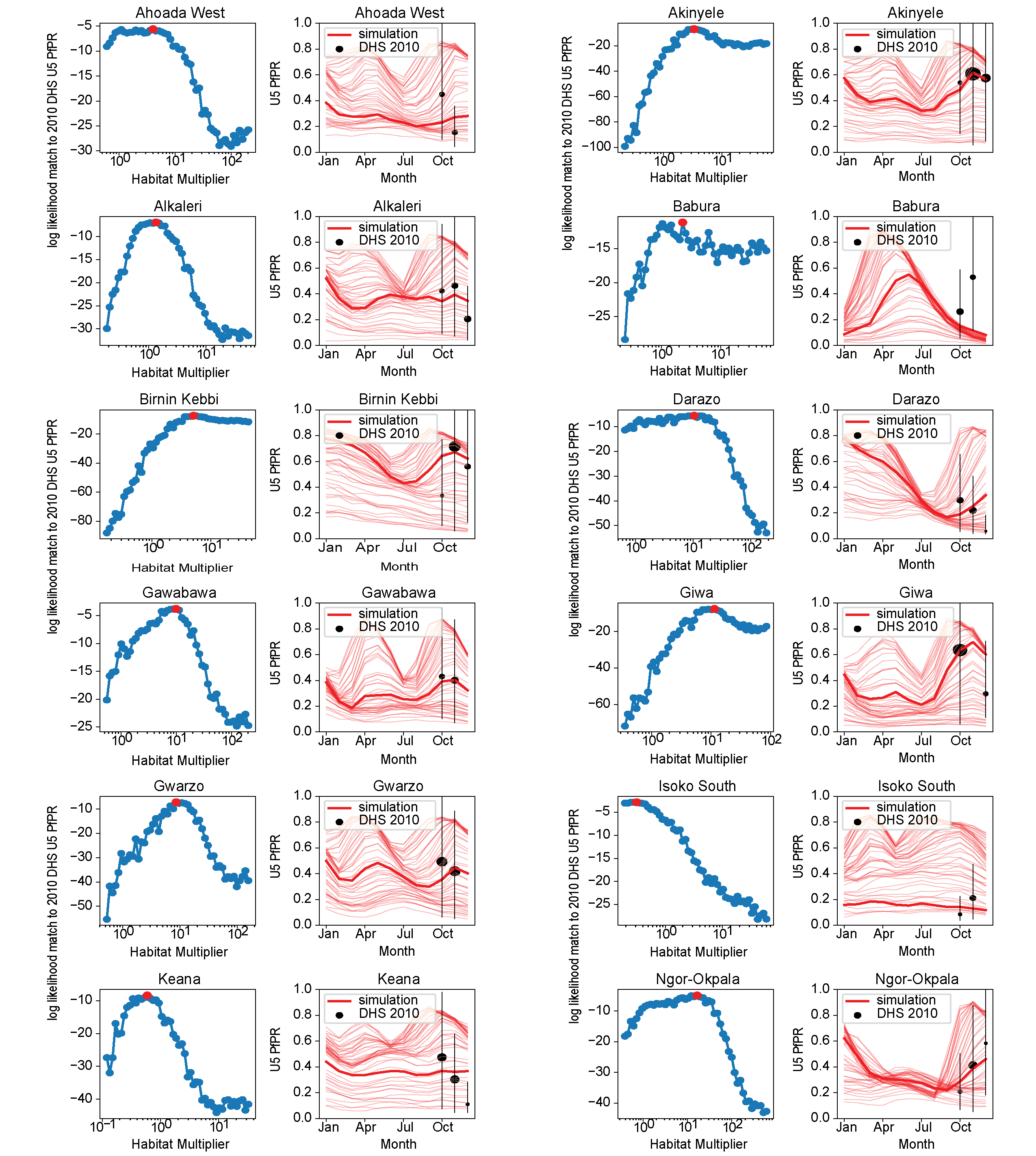


Figure S3b: Two plots are shown for each archetype with archetype names at the top of each plot. The left plot is the larval habitat multiplier (LHM) and likelihood evaluation against archetype U5 *Pf*PR 2010 MIS. The red dot is maximum likelihood estimate of LHM. The right plot is the simulated U5 *Pf*PR within each archetype compared with monthly U5 *Pf*PR from the 2010 MIS. The thick red line indicates the best match while thin red lines show *Pf*PR under other larval habitat scale factors. Each line is the mean of 10 stochastic realizations. 12 out of the 22 archetypes are shown here, remainder are shown in Fig S3b.


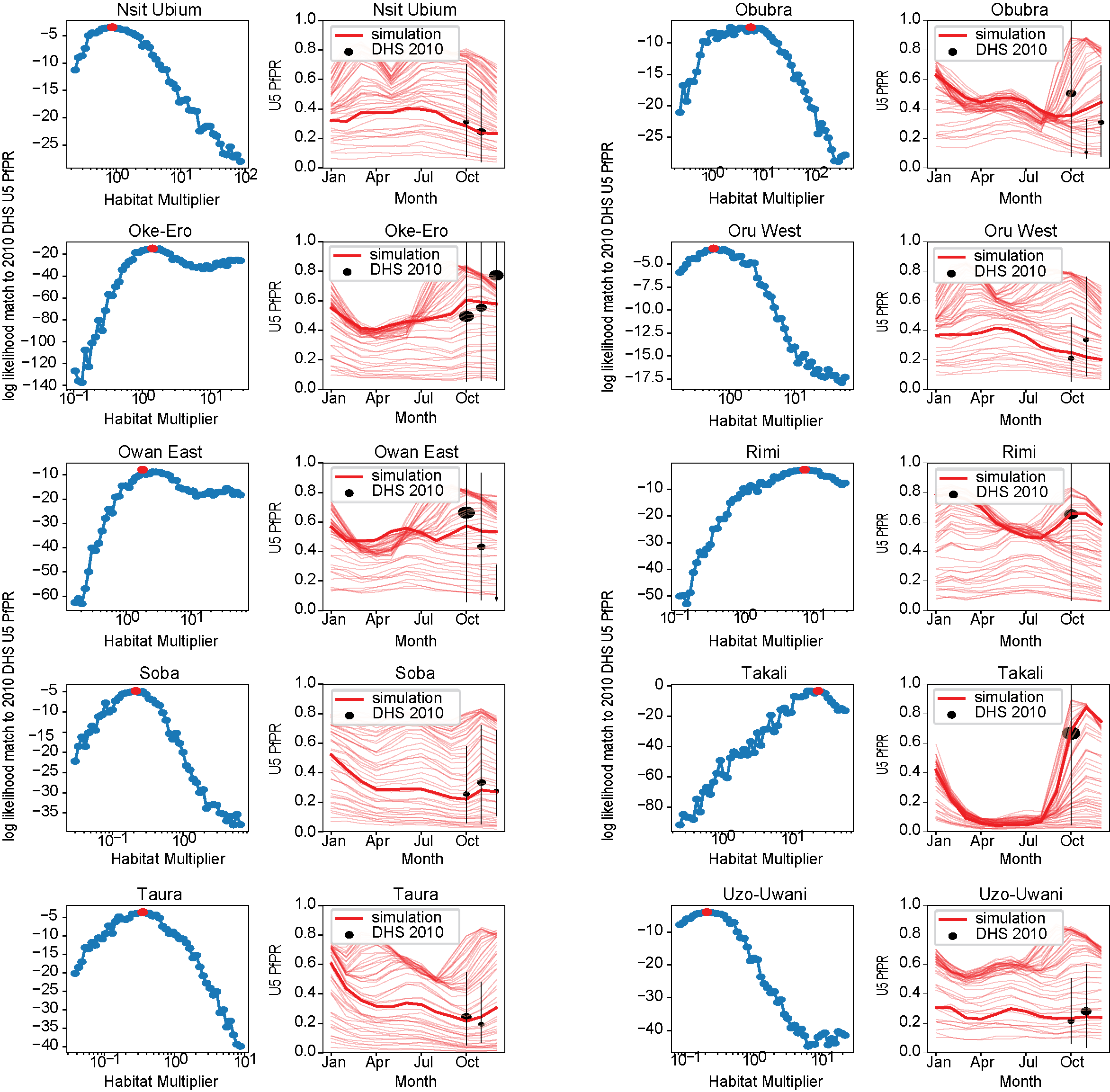


Figure S3b. Continuation of Fig S3a.

# Parameterizing intervention coverage

Intervention coverage at the LGA-level for case management, ITN use, and IPTp was parameterized using data from the Demographic and Health Surveys (DHS), the Malaria Indicator Surveys (MIS), and programmatic data.

- Whether individuals received effective case management for uncomplicated malaria was assessed from a DHS/MIS question inquiring whether a child with fever within the two-week period before the survey received artemisinin combination therapy (ACT) (Question item name in DHS data dictionary: ML13e).
- ITN use for all ages was assessed from DHS/MIS responses to the question on the type of bednet that an individual slept under the night before the survey (Question item name in DHS data dictionary: HML12).
- Program data was used to estimate ITN coverage among pregnant women who attended antenatal care (ANC) in 2018 by dividing the number of nets distributed at ANC by the estimated number of pregnancies (Figure S4).
- Receipt of IPTp was assessed using two DHS/MIS questions: 1) Whether parous women took SP/Fansidar during their most recent pregnancy (Question item name in DHS data dictionary: M49A); and 2) The number of times respondents to question 1 took Fansidar during pregnancy (Question item name in DHS data dictionary: ML1).


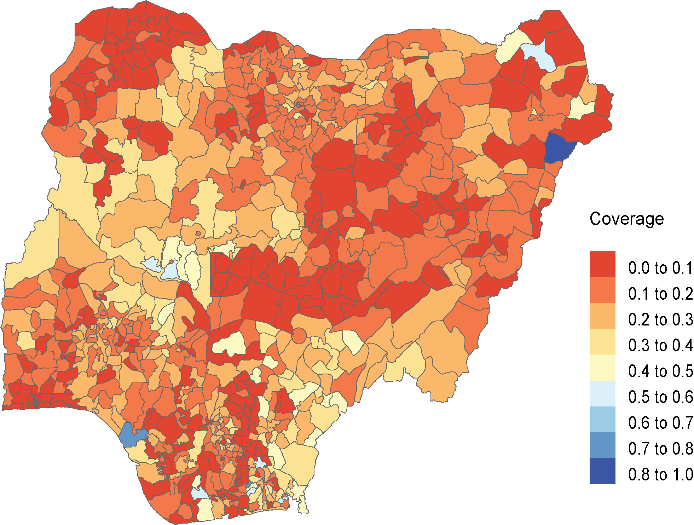


Figure S4: ITN coverage among pregnant women attending ANC in 2018

# Parametrizing the ITN model

ITN efficacy in EMOD is determined by two parameters: killing and blocking rates. ITN killing rate is the probability that a treated net kills a mosquito given an attempted indoor feed on a protected human, whereas ITN blocking rate is the probability that a net blocks an attempted indoor feed on a protected human. ITN killing and blocking parameters were adjusted to account for reported pyrethroid resistance in predictions of the impact of ITNs on morbidity and mortality indicators at the LGA-level [10]. Widespread *A. gambiae* complex resistance to 4 pyrethroids – deltamethrin, permethrin, λ-cyhalothrin, α-cypermethrin – has been observed in West and East Africa from 2005 – 2017 [11]. Previous parameterization of ITN efficacy did not account for resistance and was based on calibration to incidence reduction in older ITN clinical trials [12,13].

## ITN blocking rate

In EMOD, the blocking rate is the probability that an ITN successfully blocks a blood-feeding attempt by a mosquito. ITN blocking rate was estimated using the following equation:

$$Blocking rate= 1- \frac{x}{0.81}$$

where $x$ is the blood-feeding rate for a treated net and 0.81 is the blood-feeding rate for an untreated net with 6 holes [14], which was assumed to approximate a lack of net. The blood-feeding rate for treated nets was estimated as 0.38 through a literature review [15–17], resulting in a blocking rate of 0.53. The 0.53 value for blocking rate was used for all simulations with pyrethroid nets, including during the kill rate calibration process, regardless of local insecticide resistance.

Blocking rates for non-pyrethroid nets were assumed to be higher than those of pyrethroid nets and ranged between 0.61 – 0.94 depending on the level of insecticide resistance.

## ITN killing rate

### *Permethrin (pyrethroid) nets*

Since resistance to pyrethroids is spatially varying across Nigeria and has increased through time, yet primary measurements of resistance are not universally available, we used Malaria Atlas Project (MAP) estimates of pyrethroid resistance at the LGA level between 2010 and 2017 to inform the effect size of pyrethroid nets distributed in the simulations [11]. MAP used insecticide resistance surveillance data to generate annual pixel-level estimates of permethrin bioassay mortality rates, which we aggregated to LGA-level. To convert bioassay mortality rates into ITN kill rates of appropriate magnitude in EMOD, we use a previously published statistical model [18] to estimate experimental hut kill rates from the bioassay mortality rates, then rescaled the experimental hut kill rates into input parameters for EMOD using ITN trial data from Burkina Faso.

1. We generated a simulation model of the control arm of the Olyset Duo bednet trial that took place in Banfora health district in 2014 [19] (“Banfora trial” model). We had previously constructed a model of Banfora health district for Burkina Faso national strategic planning (“Banfora non-trial”) in an analogous process to what is described here for Nigeria. In the Banfora trial model, we distributed additional pyrethroid ITNs at 99% coverage (coverage and net type for the control arm reported in the trial paper); these ITNs were not distributed in the Banfora non-trial model.
2. Using the reported U5 incidence in the control arm of the Olyset Duo trial in Banfora (2.2 episodes per child per year) [19], we estimated the effect size of the trial’s control arm versus business-as-usual (BAU) in the rest of Banfora by calculating a relative reduction in U5 incidence. U5 incidence in BAU Banfora was estimated by taking confirmed cases in children under 5 in 2014 from Burkina’s HMIS and inflating by 1/(treatment-seeking rate in children under 5). Treatment-seeking rate was estimated at 50% based on Burkina Faso’s 2014 DHS, implying that one-half of cases are untreated and will not show up in the HMIS, hence the need to inflate confirmed cases recorded in the HMIS. This method produced an estimate that the fresh distribution of new pyrethroid nets reduced U5 clinical incidence by 12%.
3. Using the Banfora non-trial simulation model, we inferred an annual clinical incidence in children under five at 6.5 cases per child. This included both treated and untreated cases.
4. Using the Banfora trial model, we generated a suite of simulations where the initial killing rate of the pyrethroid nets varied from 0.1 to 0.9. Net usage patterns (seasonality of usage, net retention half-life) were taken from Burkina data. For each simulation, we measured the clinical incidence in children under 5 years and calculated the percent reduction from the 6.5 cases per child per year in the Banfora non-trial model.
5. We found that an initial killing rate of 0.33 in the model would generate the 12% estimated reduction in incidence from the trial data (Figure S5). This produced an anchor point for translating estimated permethrin mortality rates into initial killing rates for input to EMOD.

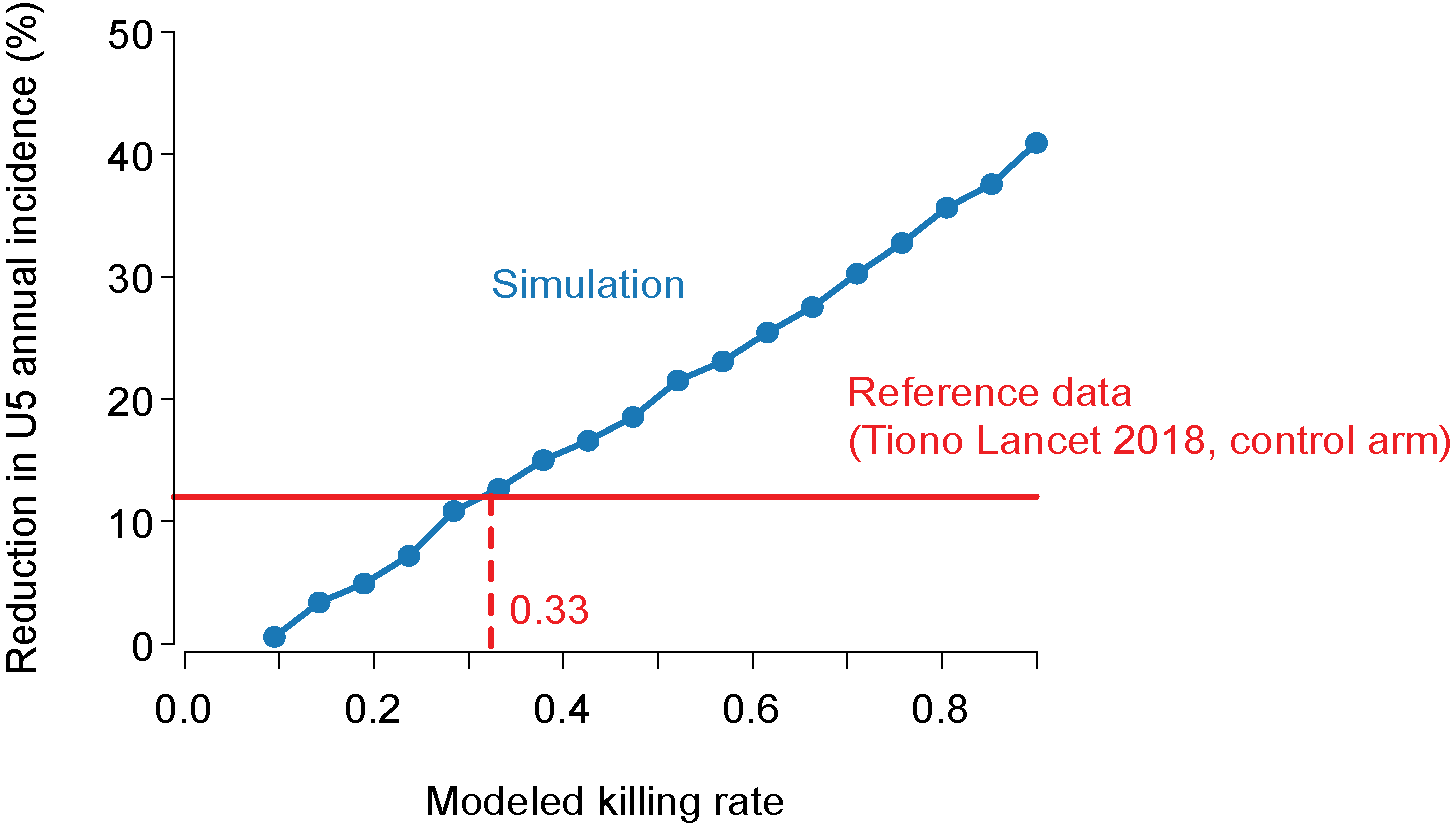


Figure S5: Estimated ITN kill rate for a 12% reduction in annual malaria incidence among children under the age of five years

1. We took the statistical relationship as defined in Churcher et al. 2016 [18], Figure 2a and Equation 2 best-fit model, and assumed the relationship between bioassay mortality and EMOD killing rate would be a linear scaled version of the relationship between bioassay mortality and experimental hut killing. We determined the scale factor by looking up the MAP estimate of permethrin mortality in Banfora in 2014 (0.24), calculating the experimental hut kill rate from the Churcher et al. model (0.40), and comparing with our fitted value of 0.33 from step 5 above. This yielded a scale factor of 0.83 to translate the Churcher et al. kill rate to EMOD kill rate (Figure S6).


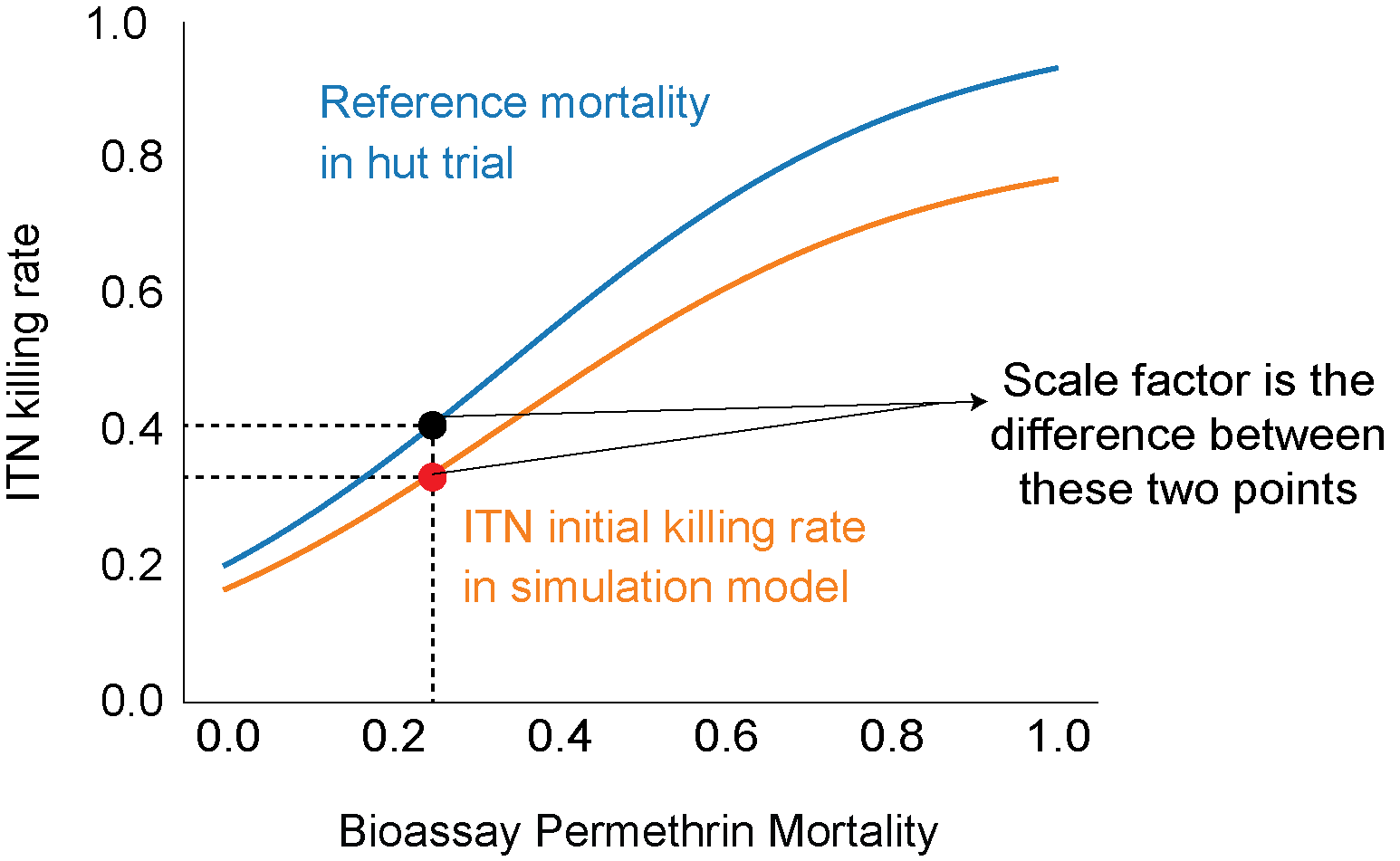


Figure S6: The relationship between permethrin bioassay mortality and ITN killing rate in the Churcher et al. model and EMOD. Scale factors was calculated by dividing the EMOD kill rate (0.33) by the kill rate from the Churcher et al. model (0.40).

1. Predicted permethrin bioassay mortality maps generated by MAP were plugged into the statistical model then rescaled to estimate ITN killing rates for individual LGAs in Nigeria for nets distributed between 2010 and 2017; nets distributed after 2017 were assumed to have kill rates corresponding to MAP’s 2017 estimate of permethrin mortality for the LGA. Permethrin net kill rates, accounting for resistance, ranged from 0.25 to 0.73, across all 774 LGAs.

### *Pyrethroid piperonyl butoxide (PBO) nets*

Parameterization of EMOD to capture the efficacy of PBO nets for individual LGAs in Nigeria followed a similar pattern as permethrin nets. Using predicted permethrin mortality data, we computed the proportion of mosquitoes killed by insecticides that contain both pyrethroids and PBO $(f)$ in a bioassay using the following equation from Churcher et al [18]:

$$logit\left( f \right)= \beta_{1}+ \frac{\beta_{2} (x- \tau)}{1+ \beta_{3} \left( x- \tau\right)}$$

where $x$ is the proportion of mosquitoes that die in a non-PBO bioassay, $\beta_{1}$is 3.41, $\beta_{2}$is 5.88, $\beta_{3}$and 0.78, and $\tau$ is 0.5. Equation 2 from [18] was also used to infer ITN killing rate for PBO nets. Combining$f$, the proportion of dead mosquitoes in a pyrethroid+PBO net, into Churcher et al.’s Equation 2, we obtained the PBO killing rate for experimental hut trials. Because there was no local clinical trial to inform the rescaling of experimental hut killing into a kill rate for EMOD, we assumed a scale factor of 0.81 such that the maximum killing rate was capped at 0.75. Kill rates for PBO nets ranged from 0.58 to 0.75 across all eligible LGAs in the national malaria strategic plans with varying levels of coverage and in the budget-prioritized plans, . Given that the initial parameterization of ITN killing rate was calibrated to 0.9 for pyrethroid nets before widespread resistance was established, we felt that 0.75 was reasonable for the maximum efficacy of PBO nets. More data from Nigeria is urgently needed to refine these estimates.

### *IG2 nets*

The Nigerian NMEP proposed to distribute Interceptor® G2 (IG2) nets, with α-cypermethrin and chlorfenapyr as active ingredients, a new type of net Due to the lack of evidence to infer the kill rate for the IG2 nets in Nigeria, we assumed a kill rate of 0.9 across the 33 LGAs where there were distribution plans. This is because we expected lower levels of resistance to these nets.

# Intermittent preventive treatment in pregnant women (IPTp)

## IPTp coverage 2010-2020

The results from the 2008, 2010, 2013, 2015, and 2018 DHS and MIS surveys were used to estimate the fraction of pregnant individuals who receive one or more IPTp doses (‘IPTp coverage’) between 2010 - 2020. For each LGA, a monotone Hermite spline was fitted using the Fritsch-Carlson method to pass through zero in 2005 and through the MIS/DHS values for years where twenty or more individuals were included in the survey within that LGA. IPTp coverage in 2019 and 2020 was assumed to be the same as the coverage in 2018. If survey results were not available for 2018 for an LGA, the 2019 and 2020 coverages were set to the IPTp coverage among all 2018 DHS participants (Figure S7).


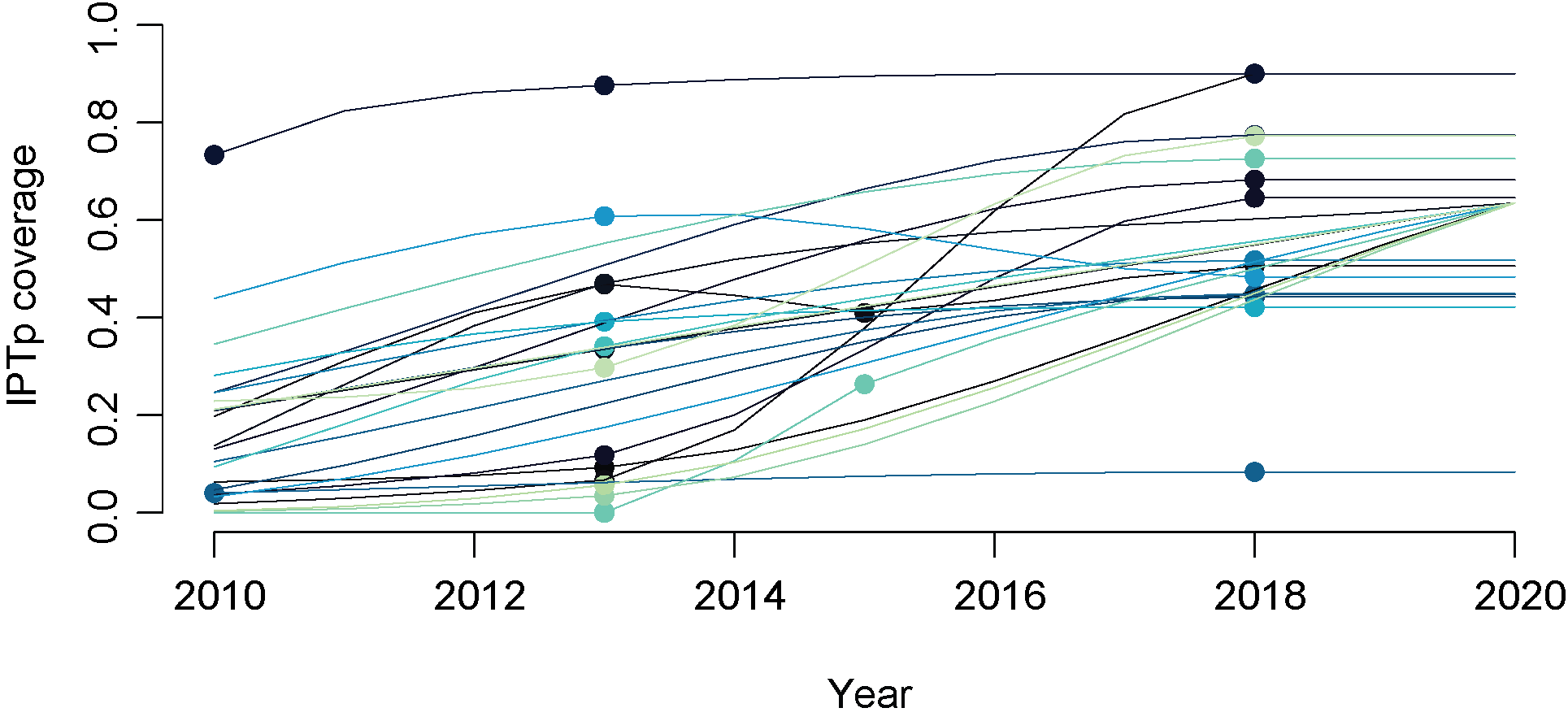


Figure S7: Fitted splines showing estimated IPTp coverage through time for a random subset of LGAs. Points show DHS/MIS data and lines show the fitted splines, with each color indicating a different LGA.

## Estimating the fraction of individuals that received different IPTp doses

To estimate the fraction of individuals who received one, two, three or more IPTp doses between 2010 and 2020, we examined DHS and MIS results (Figure S8) and observed a fluctuating pattern with increases and decreases in the fraction of individuals receiving three doses through time. The two MIS surveys in 2010 and 2015 tended to have a larger fraction of individuals receiving three or more IPTp doses than the three DHS surveys, so it seemed likely that these fluctuations result from differences between the DHS and MIS surveys or from finite survey sizes rather than from true year-to-year changes in the number of doses received. We therefore assumed a constant fraction of individuals with each number of doses through time, using the 2018 DHS values for all years because it included substantially more individuals than previous surveys.


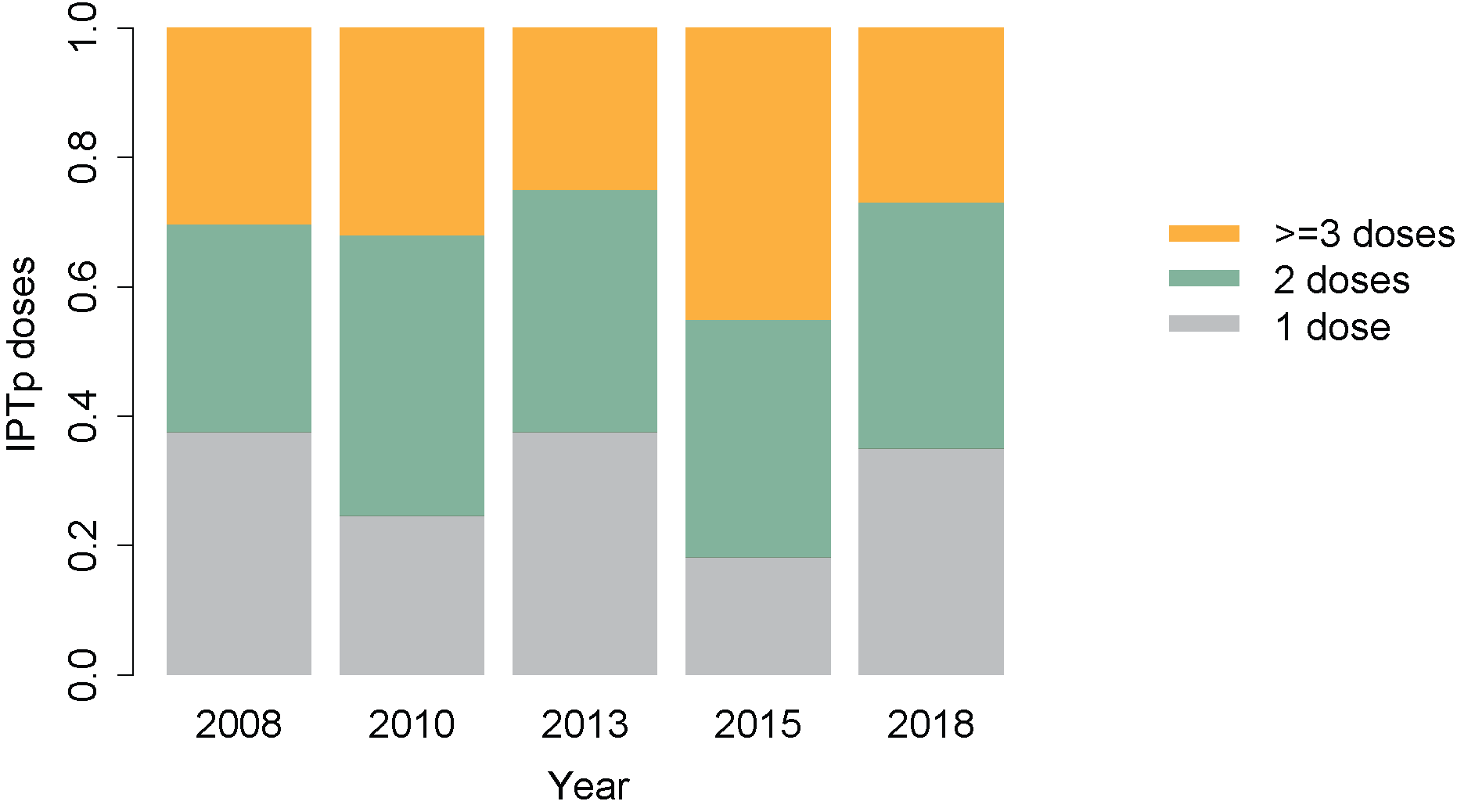


Figure S8: Fraction of IPTp-receiving individuals who reported receiving one, two, or three or more doses in each DHS (2008, 2013, 2018) or MIS (2010, 2015).

## Adjusting PfPR to account for the impact of IPTp

IPTp reduces parasite prevalence among pregnant individuals by clearing existing infections and protecting against new infections [20]. IPTp-caused parasite clearance was not simulated in EMOD. Rather, the simulation output was adjusted to account for the reduction in parasite prevalence among pregnant individuals, given a certain IPTp coverage. This adjustment only accounts for the direct effects of IPTp on reducing malaria prevalence among pregnant individuals and does not account for any indirect effects that IPTp may have on other groups by reducing transmission in the population. For example, this process ignores that 1) New infections may occur in the simulations caused by the person who should have been protected by IPTp; 2) Individuals who *should* be protected by IPTp but nonetheless get malaria in the simulations will develop more protective immunity in the simulations than they should have; 3) There ought to be a delay between when the protective effects of IPTp end and when an individual is next infected, but that delay is not included in adjusting *Pf*PR (it depends on EIR and is unlikely to make a noticeable difference).

However, given the relatively small fraction of the population receiving IPTp at a given time and the high transmission intensity in many of the simulated regions, the impact of IPTp on prevalence in non-pregnant groups is likely to be relatively small.

To adjust the *Pf*PR among pregnant individuals who take IPTp, we made a rough estimate, informed by literature [20], that taking one IPTp dose protects against infection for ten weeks and that each subsequent dose, up to three doses, adds an additional four weeks of protection. Assuming a 36-week pregnancy, the fraction of the pregnancy that is unprotected for individuals who take 1, 2, and 3 IPTp doses is estimated to be 0.722, 0.611, and 0.5, respectively. The adjusted PfPR among pregnant individuals in a particular age group was thus calculated as

*adjusted_PfPR* = *original_PfPR* * (f_0_ * 1 + f_1_ * 0.722 + f_2_ * 0.611 + f_3_ * 0.5)

where f_0_, f_1_, f_2_, and f_3_, represent the fraction of pregnant individuals who receive zero, one, two and three IPTp doses, respectively.

The impact of IPTp on maternal and infant mortality is discussed in the “Estimating malaria mortality” section.

# Intermittent Preventive Treatment in Infants (IPTi)


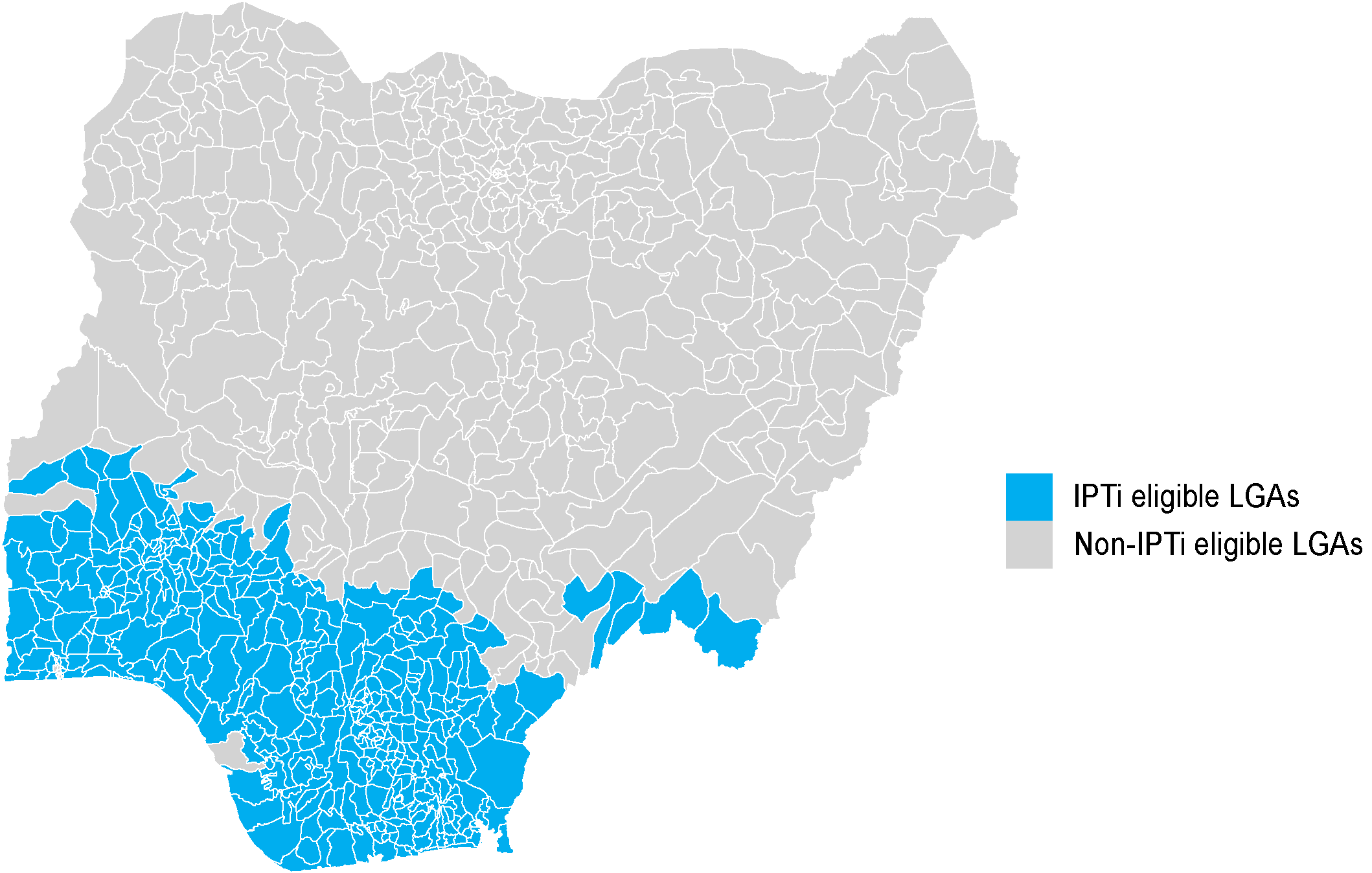


Figure S9: LGAs designated as eligible to receive IPTi.

## Estimating likely IPTi coverage through historical vaccine coverage

374 LGAs were deemed IPTi-eligible based on SMC ineligibility and transmission intensity (EIR >10) [21] (Figure S9). IPTi, if implemented, would be administered through the Expanded Programme on Immunization (EPI) in Nigeria. The DHS captures vaccine coverage based on vaccine record cards, or from verbal recall from the mothers, and the 2018 DHS survey was used to inform the assumptions on feasible IPTi coverage. IPTi would be administered at age of around 10 weeks, 14 weeks, and 9 months, aligning with the schedule for pentavalent DTP vaccine doses 2 and 3 and measles vaccine. The reported pentavalent DTP vaccine coverages for the doses 1, 2 and 3 per household cluster were aggregated per LGA and average of the three doses taken. Missing values were replaced with the state estimate (n= 47 LGAs within the IPTi eligible areas) (Figure S10).

We considered the impact of IPTi in Scenario 2 with coverage targets at or above 80%. Given that IPTi had never been implemented in Nigeria but was expected to be co-administered with EPI vaccines, ‘historical coverage levels’ were assumed to be the same as that of the pentavalent vaccines from the 2018 DHS survey. Increases in coverage were applied to these “historical” coverage levels.


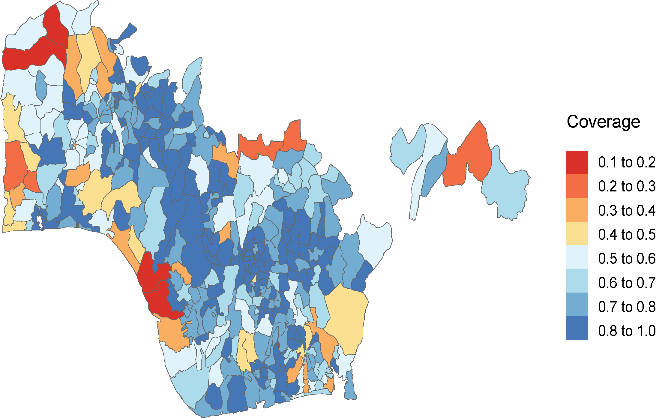


Figure S10: Mean DTP1-3 vaccine coverage per LGA in the 2018 DHS survey

## Adjusting prevalence, incidence and mortality for the impact of IPTi

IPTi reduces prevalence, cases and mortality in infants by clearing infections and protecting against new infections for 30 days following a dose. Sulphadoxine-pyrimethamine (SP) is the recommended drug for IPTi. Synthesized protective efficacy (PE) estimates from clinical trials conducted between 2000 and 2012 suggest PE’s ranging from 0.66 for prevalence to 0.93 for all-cause mortality [22] (Table S2). Since these estimates are for the total infant population after receiving all IPTi doses, the IPTi adjustment was done for the total infant population using aggregated IPTi coverage for all doses. The IPTi adjustment was applied after adjusting for the impact of IPTp by applying scaling factors onto the simulation outputs for the U5 age groups and for the total population. To obtain these scaling factors, we first (i) calculated the total number of infections, cases and deaths averted due to IPTi in infants, (ii) then subtracted these from the respective number in the U5 and total population and (iii) calculated relative reductions to be used as scaling factor on the IPTp-adjusted predictions.

*Table S2: Synthesized IPTi protective efficacy estimates extracted from Esu et al* [22]

| **Outcome** | **Rate ratio** | **95%CI** |
| --- | --- | --- |
| *Pf*PR | 0.66 | (0.56 - 0.79) |
| Clinical cases | 0.79 | (0.74 - 0.85) |
| Severe cases | 0.92 | (0.47 - 1.81) |
| All-cause mortality | 0.93 | (0.74 - 1.15) |

### *Malaria infections, cases and deaths averted in infants due to IPTi*

The effect of IPTi was estimated by combining the LGA-specific coverage with efficacy parameters from Esu et al [22] (Table S2) and the LGA-specific outcome predictions in infants (≤12 months old), since the adjustment was done for a complete course of IPTi rather than per dose. The annual number of malaria positives based on microscopy (PfPR in population ≤12 months of age * total population ≤12 months of age), number of malaria cases, and number of malaria deaths per LGA were adjusted for the effectiveness of IPTi between 2020 and 2030. The mean values across all stochastic realizations were used. The IPTi effectiveness adjustment for Scenario 2 was calculated as:

$${x.adj}_{k}=x_{k}-\left( x_{k}*(1-PE)*C \right)$$

With *x* the number of events per outcome (i.e. positives, cases, and deaths) from simulation output, *k* denoting the LGA, *PE* the protective efficacy per outcome separate for mean and confidence limits, and *C* the LGA-specific IPTi coverage. The number of deaths in infants was estimated based on severe cases using the approach described below in the ‘Estimating malaria mortality’ section and assuming the same case fatality and treatment seeking rates as for children U5.

### *Malaria infections, cases and deaths averted in U5 and total population*

The number of infections, cases and deaths averted in infants due to IPTi were subtracted from the respective outcomes in the U5 and total population per LGA and year. Next, the relative reduction due to IPTi was calculated as the ratio of the adjusted and unadjusted predictions, i.e. number of infections, cases and deaths accounting for events averted in infants due to IPTi, divided by number of infections, cases and deaths without accounting for events averted in infants. The calculations were done for mean as well as confidence limits of the IPTi PE’s.

### *Use of relative reductions per age group as scaling factors for IPTi effectiveness*

To combine the adjustment for effectiveness of IPTi with the adjustment for IPTp effectiveness in children U5 and the total population, the calculated relative reductions were used as scaling factors and applied on the predictions after these were adjusted for IPTp. However, as the intervention targets were infants, the effect on older age groups and the total population was negligible.

# Estimating malaria mortality

Several sources of mortality were included in the analyses: 1) mortality from severe malaria, 2) maternal mortality from severe anemia caused by malaria in pregnancy, and 3) infant mortality resulting from low birth weight that occurred due to malaria in pregnancy.

## Mortality from treated and untreated severe malaria

The estimated case fatality rates (CFR) for treated severe malaria varies widely between studies and study sites, and most studies that examine the fatality rates among hospitalized severe cases look only at hospitalized children [23–28]. Our estimate of CFR was based on a published analysis of clinical trial data from nine African countries comparing outcomes of children <15 years old with severe *P. falciparum* malaria treated with artesunate or quinine, where the aggregated CFR across eleven study sites was 9.7% [16]. The mortality rates from across the sites included in [25] largely span the range of mortalities reported in [23,24,27,28].

We found limited data describing the mortality rates for untreated severe cases in the literature, and the criteria used to ascribe whether a set of symptoms constitutes severe disease varies across studies. In [29], the results from [30] and [31] were combined to estimate an 82% protective efficacy of treatment of severe *P. falciparum* malaria in reducing mortality in children by comparing the CFRs with effective treatment versus with treatment failure due to chloroquine resistance. In the hospitals included in the studies, which offered a high standard of care, the CFR among effectively treated children was around 3.4% and the estimated CFRs for children not receiving effective treatment were 13.1%-21.1%. However, assuming a 9.7% CFR for treated severe cases in our simulations, an 82% protective efficacy would yield an untreated CFR of:

$$CFR for untreated severe malaria=\frac{CFR for treated severe malaria}{1-protective efficacy of treatment for severe malaria}$$

$$=\frac{9.7\%}{1-0.82}$$

$=53.9\%$ .

Similarly, [32] reported a 33% CFR for hospitalized children who received chloroquine or no antimalarials in an area with chloroquine-resistant parasites.

In [33], the authors estimated the odds ratio for severe case fatality without versus with hospitalization as around 2. If we assume that hospitalization equates with treatment and use the 9.7% CFR for treated severe cases, we estimate an untreated CFR for our simulations as follows:

$$Odds ratio=2=\frac{\left( \frac{p_{u}}{1-p_{u}} \right)}{\left( \frac{p_{t}}{1-p_{t}} \right)}= \frac{\left( \frac{p_{u}}{1-p_{u}} \right)}{\left( \frac{0.097}{0.903} \right)}$$

$\to p_{u}= \frac{2* \frac{0.097}{0.903}}{\left( 1+2* \frac{0.097}{0.903} \right)}= 0.177$ .

Where the probability of death for severe, untreated cases is *p_u_* and the probability of death for severe, treated cases is *p_t_*.

For the analysis results reported in the main text, we used an intermediate severe, untreated CFR value of 33%, but we also repeated the analyses for CFRs of 53.9% and 17.7% and saw very similar patterns of mortality rates relative to the business as usual scenario. We assume all CFRs are age-independent and that all deaths are preceded by an episode of severe malaria. Incidence of severe malaria in EMOD is calibrated to data on severe disease from [34] following the method in [35].

## Mortality from severe maternal anemia attributable to malaria in pregnancy

Individuals with malaria in pregnancy are at a higher risk of severe malaria and malaria-attributable mortality than their non-pregnant counterparts [36–40]. Much of this increased risk comes from severe anemia, particularly in malaria-infected first or second pregnancies [36,37,41]. For our simulations, we assumed that without IPTp, there is a 5.7% probability that malaria during a first or second pregnancy results in malaria-attributable severe maternal anemia [41–43]. We assumed that the probability of severe anemia due to malaria in pregnancy is reduced by 39% in individuals taking IPTp during pregnancy (39% from [44], 38% from [41], 49.5% for severe and non-severe anemia reported in [45]) and that the protection is the same for all dose numbers [44]. The number of simulated individuals with malaria-attributable severe anemia (*N_anemia_*) in a given month was thus estimated as:

$$N_{anemia}= N_{births}*m* \left( \left( f*0.057*\left( 1-0.39 \right) \right)+ \left( \left( 1-f \right)*0.057 \right) \right)$$

Here, *N_births_* is the number of first or second births expected to occur in the simulated population, which is calculated as the product of the simulated population size, the monthly per-capita birth rate of 0.0028 [46], and the fraction of all pregnancies that are the mother’s first or second pregnancy (estimated as 0.36). The parameter *m* represents the fraction of simulated individuals between 15-30 years of age who were infected with malaria during the previous nine months of the simulation, and *f* is the fraction of pregnant individuals who received at least one dose of IPTp.

We assumed that individuals with malaria-attributable severe anemia cases have the same treated and untreated CFRs as severe malaria in the general population.

## Mortality from low birth weight attributable to malaria

We calculated the number of births with malaria-attributable low birth weight (mLBW) and mLBW-related mortality among individuals infected with malaria during pregnancy. The risk of mLBW appeared substantially greater in first or second pregnancies [41,47,48], and this is likely especially true in regions with high transmission intensity. We assume that malaria infection during a first or second pregnancy increases the risk of a low birth weight (LBW) infant by 0.12. This value comes from an estimate that there is around twice the risk of an LBW infant if the mother is infected during pregnancy (values from the literature range from around 1.5 to 3 [38,41,42,49–51]) and the estimate that the probability of an LBW infant if there is no malaria infection is around 0.12 [51]. The risk of death in infants with low birth weights is estimated to be 3.2 times higher than infants of normal weight [49]. A similar value as was reported in [42], though [52] saw a 4 times higher risk and [53] reported a hazard ratio of 10.16 for death in LBW infants. We assumed that the probability of malaria-attributable death in a mLBW infant was 0.148, calculated using the low-birth-weight prevalence in a population [54], the estimated infant death rate for that population [55], and the relative risk of death in low-birth-weight infants.

An individual who receives IPTp during pregnancy is expected to have a reduced risk of delivering a mLBW infant relative to an unprotected mother, with the effect size dependent on the number of IPTp doses. IPTp may reduce the probability of mLBW by preventing individuals from becoming infected with malaria and/or by ending an infection before infant growth has been substantially affected. Based on the rates of placental parasitemia in Burkina Faso among primigravidae and secundigravidae who received 0, 1, 2, or 3 doses of IPTp during pregnancy [47] we assumed the risk of placental parasitemia with 1, 2, and 3 doses of IPTp, relative to no IPTp, to be 0.74, 0.45, and 0.36, respectively. Even individuals with placental parasitemia who take IPTp may have some reduced risk of mLBW due to the more rapid clearance, although the value of this parameter is unknown. Combining the estimates form [47] with the reduced risk from shortened infections, we estimate the risk of mLBW among malaria-exposed individuals with 1, 2, or 3 doses of IPTp relative to malaria-exposed individuals who did not receive IPTp as 0.67, 0.41, and 0.32, respectively. For comparison, the relative risk of LBW for 2 versus ≥3 IPTp doses was examined in a 2013 meta-analysis by [56], where the risk of LBW given three or more IPTp doses was estimated to be 80% of that with two IPTp doses. This value seems reasonably consistent with the 0.41 versus 0.32 risks used here given that studies included in the meta-analysis were conducted in holoendemic or hyperendemic regions, so many of the individuals were likely exposed to malaria during pregnancy. However, we note that there is high uncertainty associated with these estimates.

# Intervention scenarios and predictions

Visualization and tables of LGA-level inputs and predictions at the state and national level for individual intervention scenarios can be accessed through this shiny app.

## Case management (CM) coverage in intervention scenarios

Increases in CM coverages per LGA in Scenarios 2, 3 and 4 using a beta regression model fitted to archetype-level 2013, 2015, and 2018 DHS/MIS data. Predicted average yearly change in coverages from 2013 to 2018 were computed by archetype. In Soba archetype where the average yearly change in CM was negative, the value from the Isoko archetype, which had the smallest positive average yearly change was substituted. CM coverage values for each LGA were additively scaled using their archetype values according to the scenario coverage trajectory and ceiling.

For Scenario 2, which had an 80% target, a minimum yearly increase value needed to reach the target by 2030 was computed per LGA accounting for the coverage starting values and the average yearly increase values from the beta model. For Scenario 3 and 4 where coverages were expected to increase at the same rate as historical values, the beta model mean yearly increase values and their multiples was directly added to the starting CM coverages by LGA. Figure S11 shows the beta regression model fit to individual archetype data.


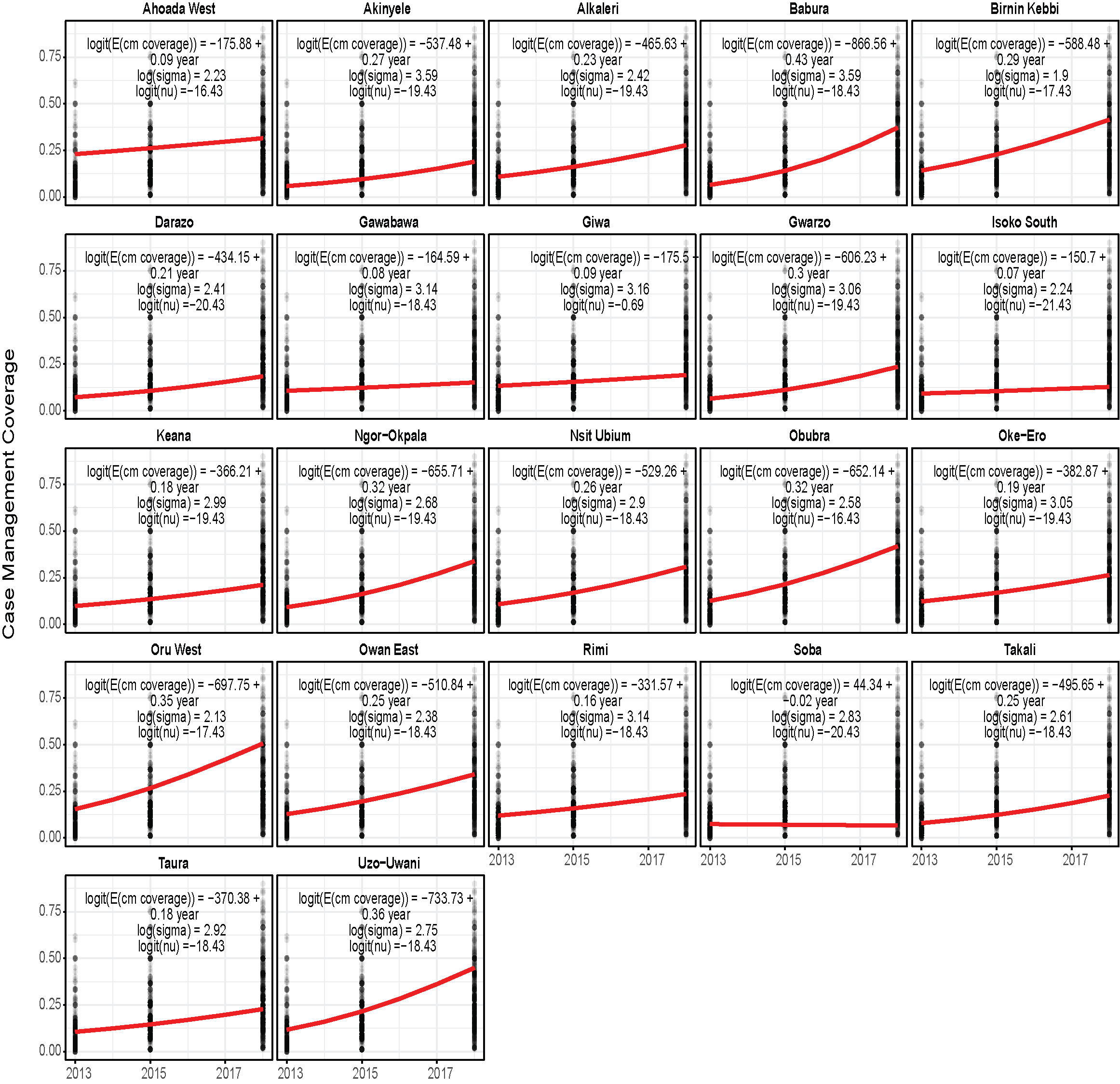


Figure S11 Archetype level scatterplot highlighting beta-regression model equations used to compute predicted average yearly change CM coverages per archetype. Annual case management is the proportion of children with fever in the two weeks period prior to the survey that received an ACT.

# Validation

Simulated incidence (treated uncomplicated cases and treated cases) was validated by comparison with state-level health facility data from 2014 – 2018 via the Rapid Impact Assessment project. Comparisons were made using a cross-correlation function (CCF). The CCF at the time lag zero is a measure of the contemporaneous correlation or the linear relationship between the two timeseries. Since the health facility data was constructed from a subset of public facilities in Nigeria, incidence values computed for years 2014 to 2018 was lower than simulated values by at least three orders of magnitude and had to be rescaled to enable visual comparison of trends. Visual comparisons and the CCF estimates at different time lags are presented in Figures S12 – S21.

## Incidence comparison plots and cross-correlation plots by state


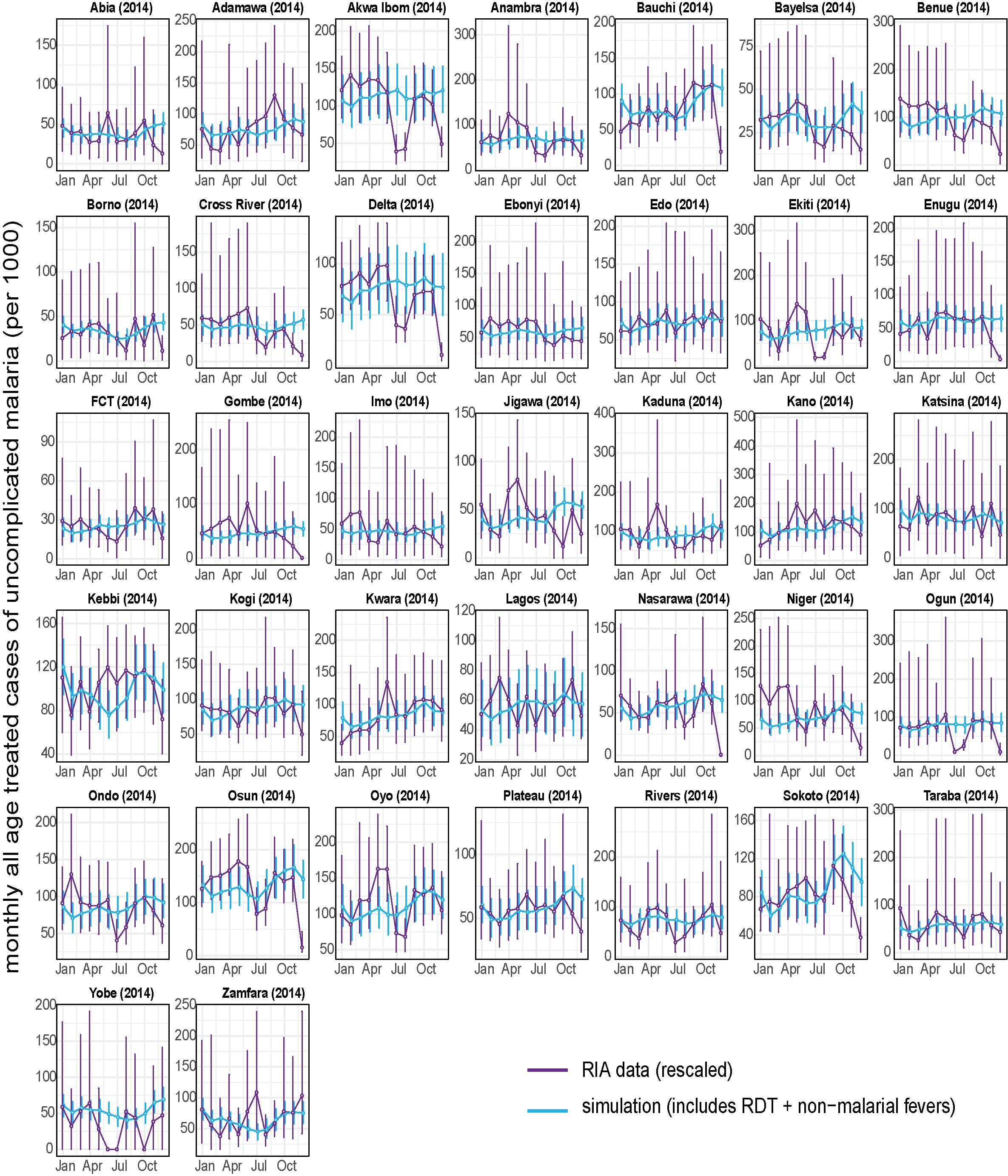


Figure S12: Malaria seasonality in routine health facility data and simulation for 37 Nigerian states in 2014. Incidence values in the health facility data were scaled by the median relative difference between the simulation and RIA data by state. Vertical purple horizontal lines are 95% confidence intervals for the RIA data. Vertical blue lines are the ranges of the simulations from 5 seed runs.


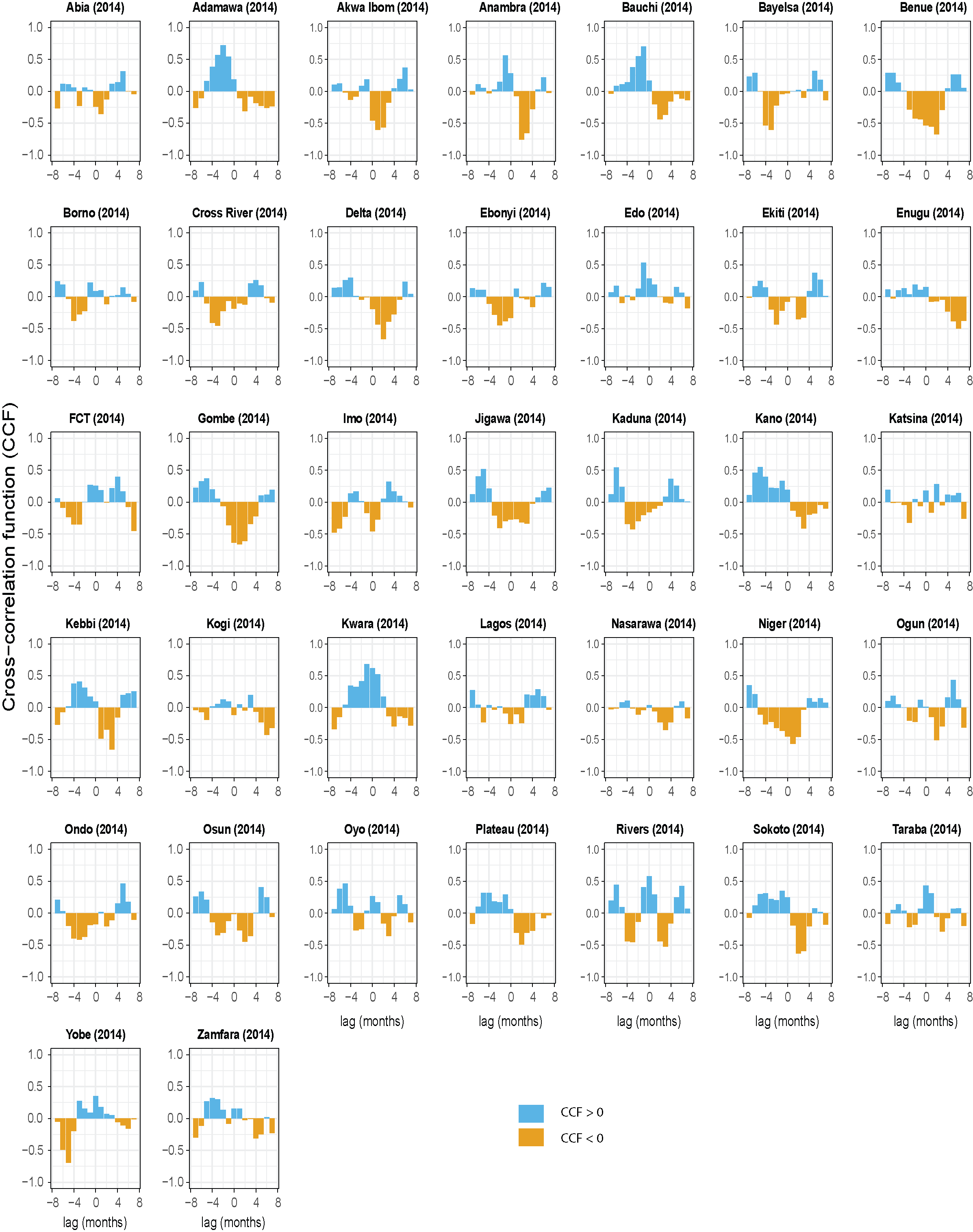


Figure S13: Comparison of DHIS2 and simulation seasonality trends in 2014 with a cross-correlation function (CCF). CCF at the time lag zero is a measure of the contemporaneous correlation or the linear relationship between the two time series.


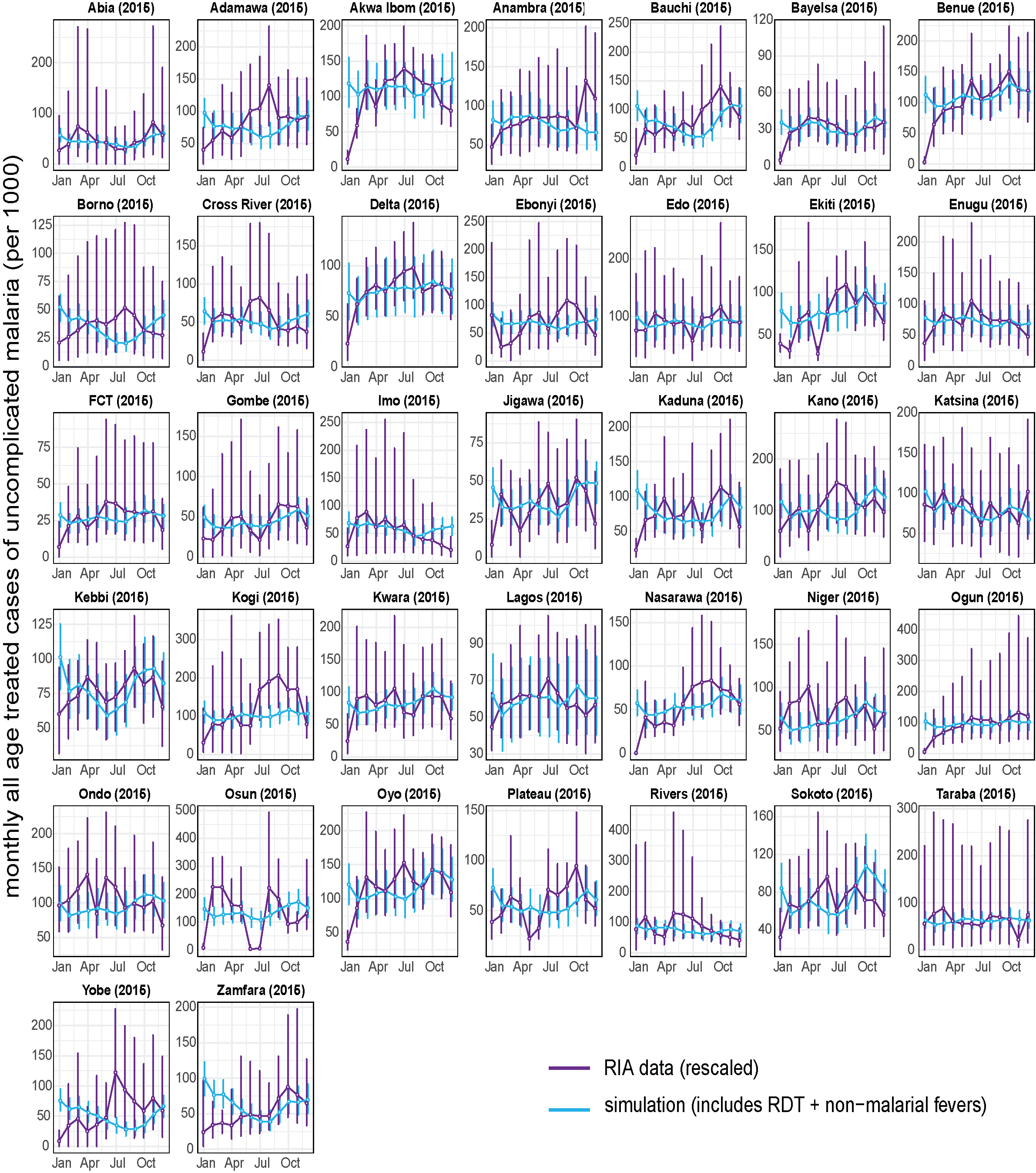


Figure S14: Malaria seasonality in routine health facility data and simulation for 37 Nigerian states in 2015. Incidence values in the health facility data were scaled by the median relative difference between the simulation and RIA data by state. Vertical purple horizontal lines are 95% confidence intervals for the RIA data. Vertical blue lines are the ranges of the simulations from 5 seed runs.


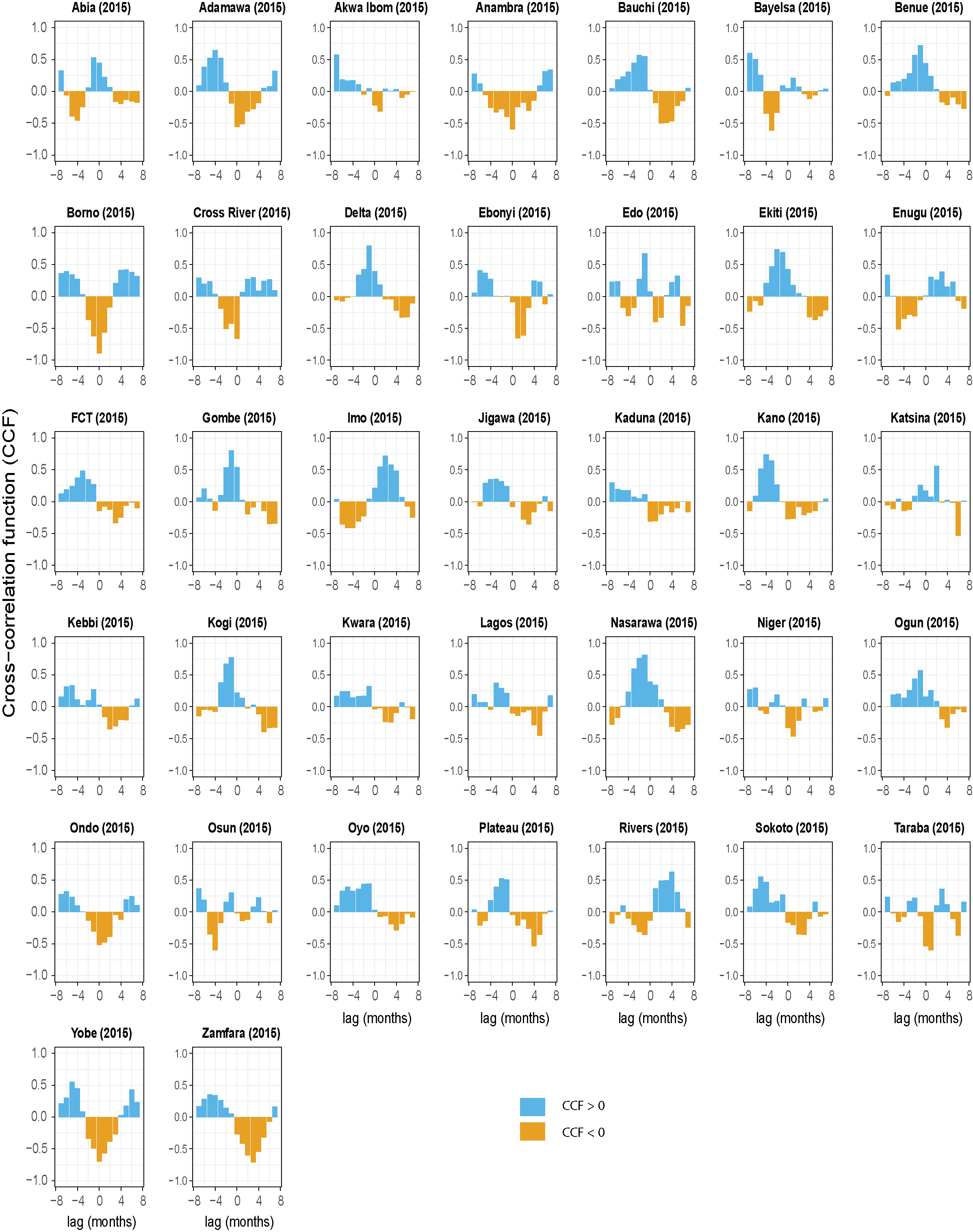


Figure S15: Comparison of DHIS2 and simulation seasonality trends in 2015 with a cross-correlation function (CCF). CCF at the time lag zero is a measure of the contemporaneous correlation or the linear relationship between the two time series.


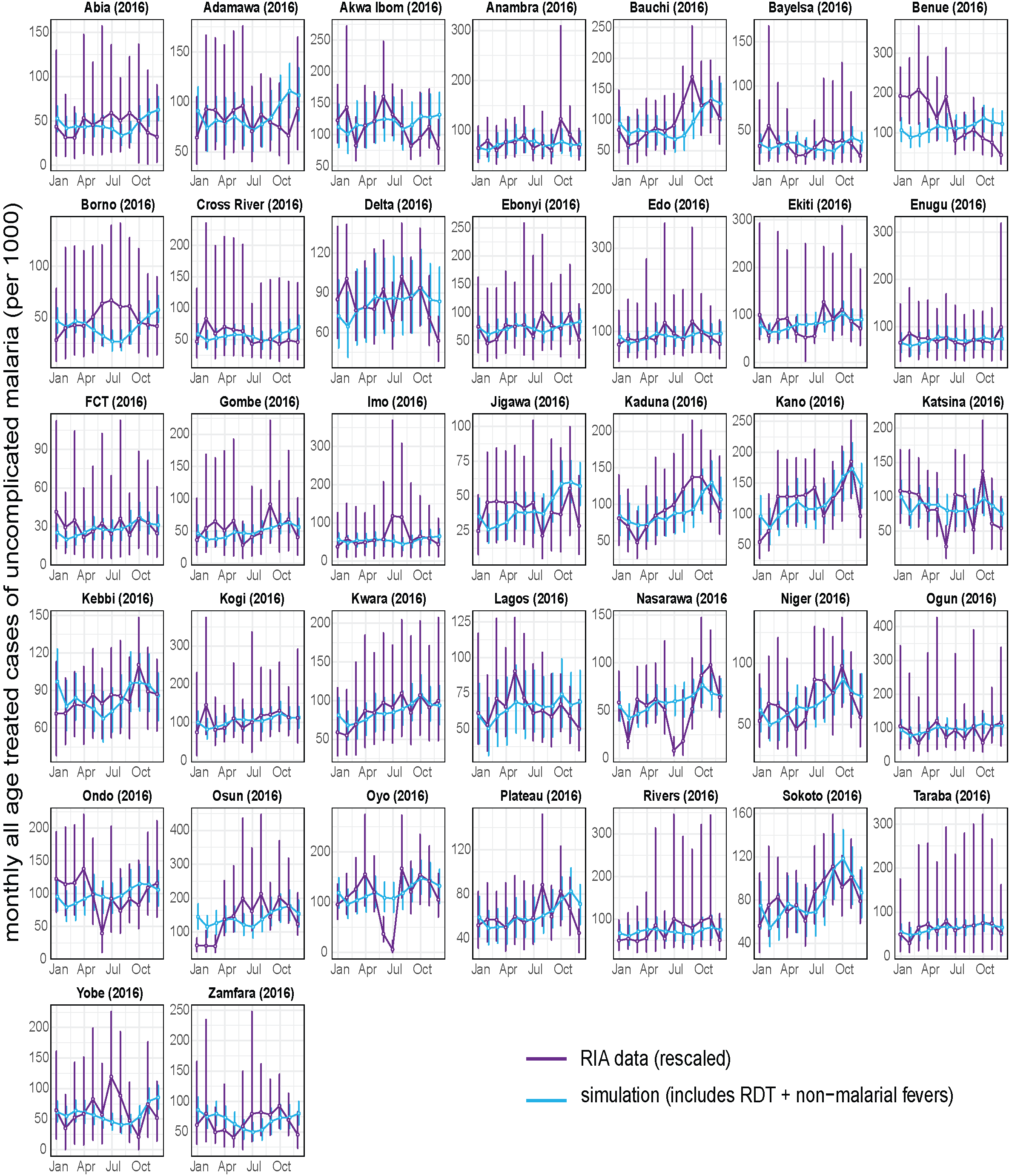


Figure S16: Malaria seasonality in routine health facility data and simulation for 37 Nigerian states in 2016. Incidence values in the health facility data were scaled by the median relative difference between the simulation and RIA data by state. Vertical purple horizontal lines are 95% confidence intervals for the RIA data. Vertical blue lines are the ranges of the simulations from 5 seed runs.


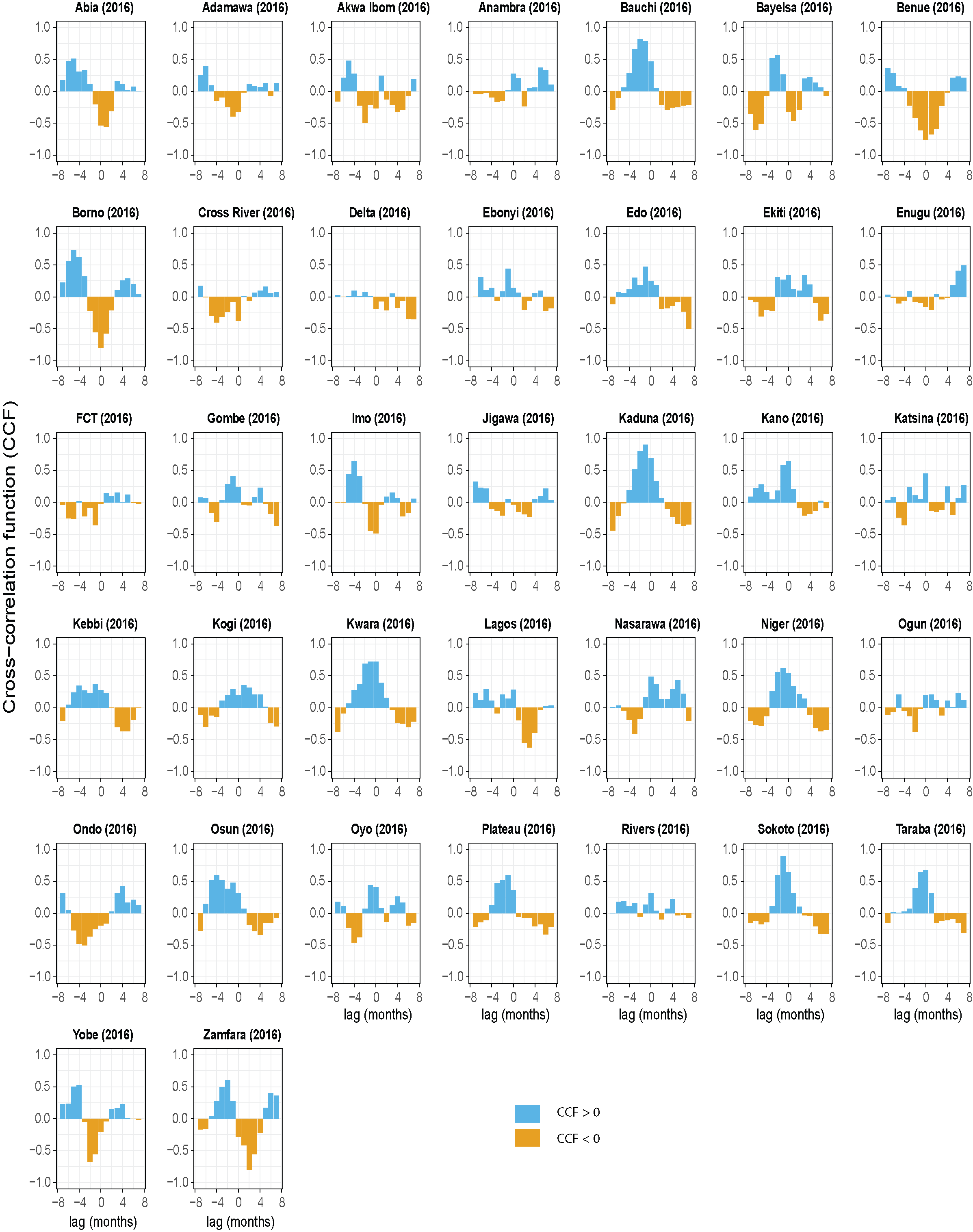


Figure S17: Comparison of DHIS2 and simulation seasonality trends in 2016 with a cross-correlation function (CCF). CCF at the time lag zero is a measure of the contemporaneous correlation or the linear relationship between the two time series.


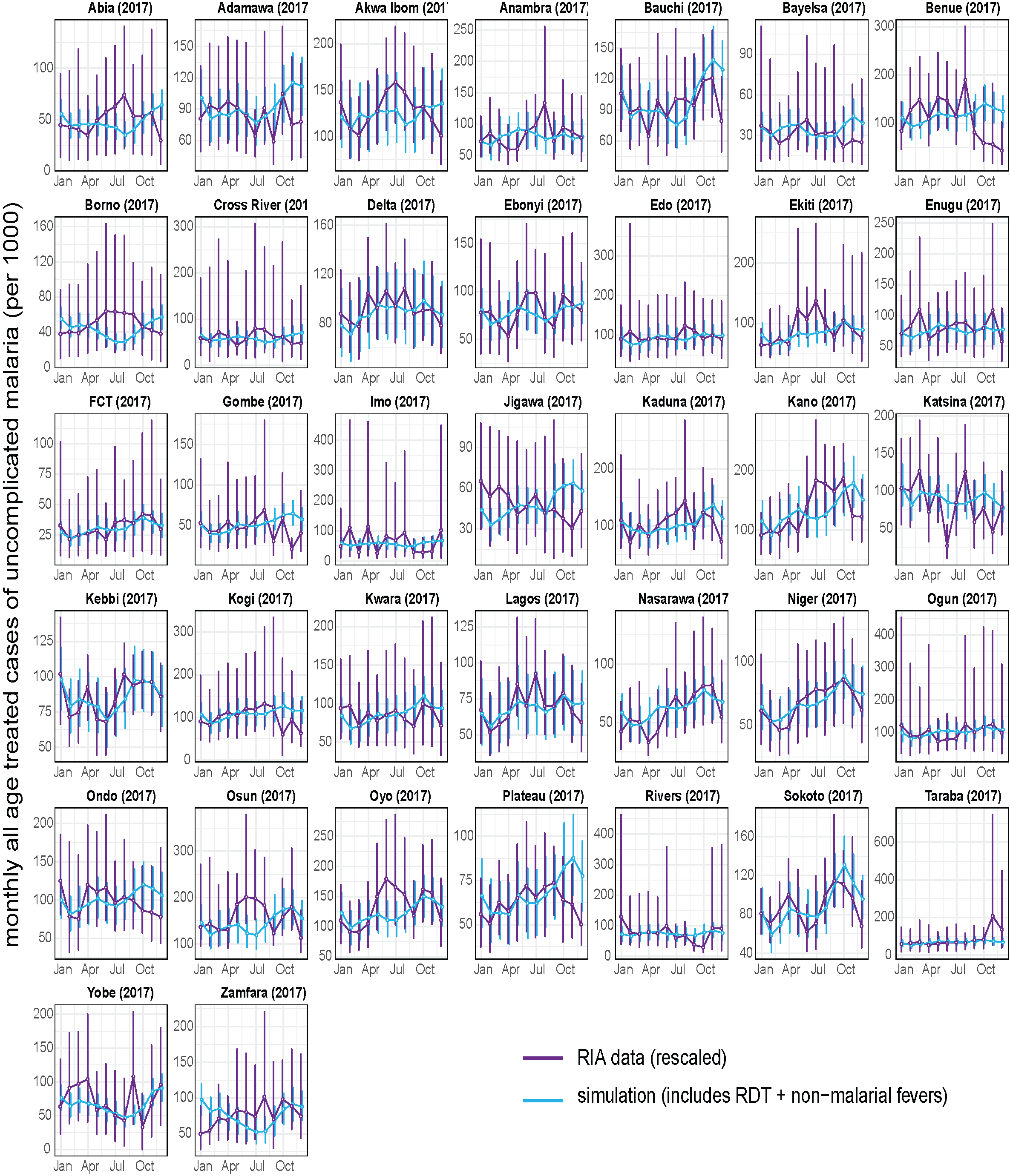


Figure S18: Malaria seasonality in routine health facility data and simulation for 37 Nigerian states in 2017. Incidence values in the health facility data were scaled by the median relative difference between the simulation and RIA data by state. Vertical purple horizontal lines are 95% confidence intervals for the RIA data. Vertical blue lines are the ranges of the simulations from 5 seed runs.


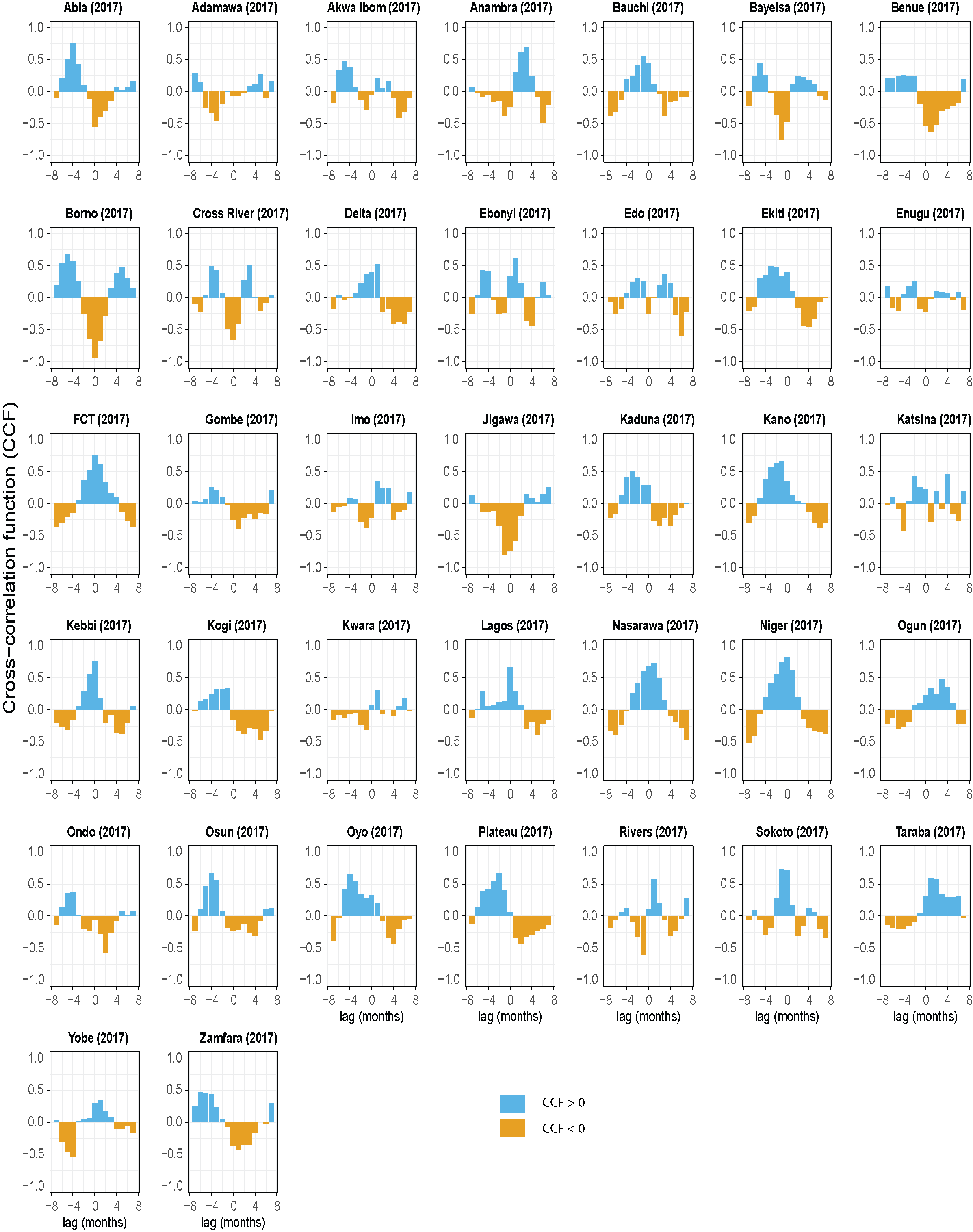


Figure S19: Comparison of DHIS2 and simulation seasonality trends in 2017 with a cross-correlation function (CCF). CCF at the time lag zero is a measure of the contemporaneous correlation or the linear relationship between the two time series.


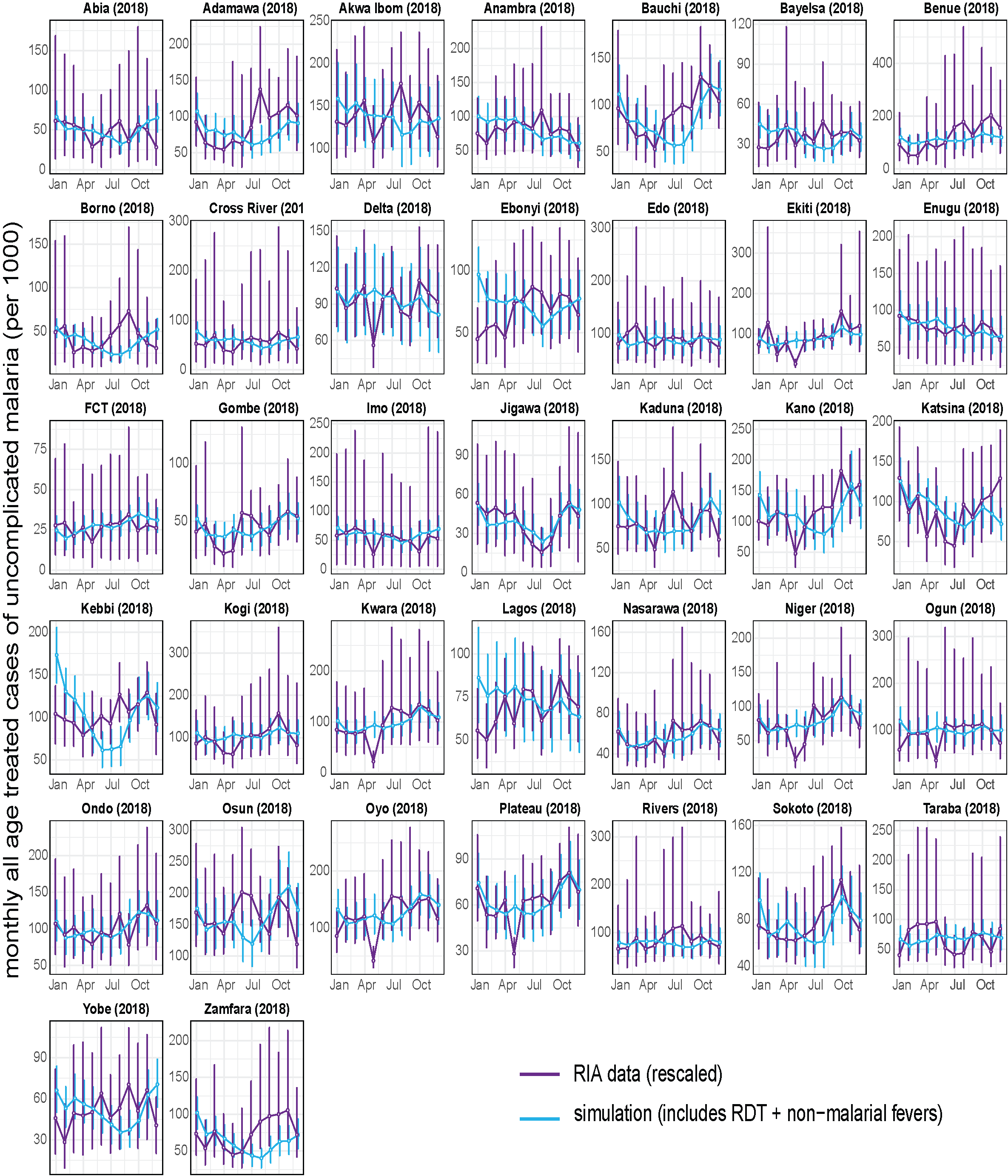


Figure S20: Malaria seasonality in routine health facility data and simulation for 37 Nigerian states in 2018. Incidence values in the health facility data were scaled by the median relative difference between the simulation and RIA data by state. Vertical purple horizontal lines are 95% confidence intervals for the RIA data. Vertical blue lines are the ranges of the simulations from 5 seed runs.


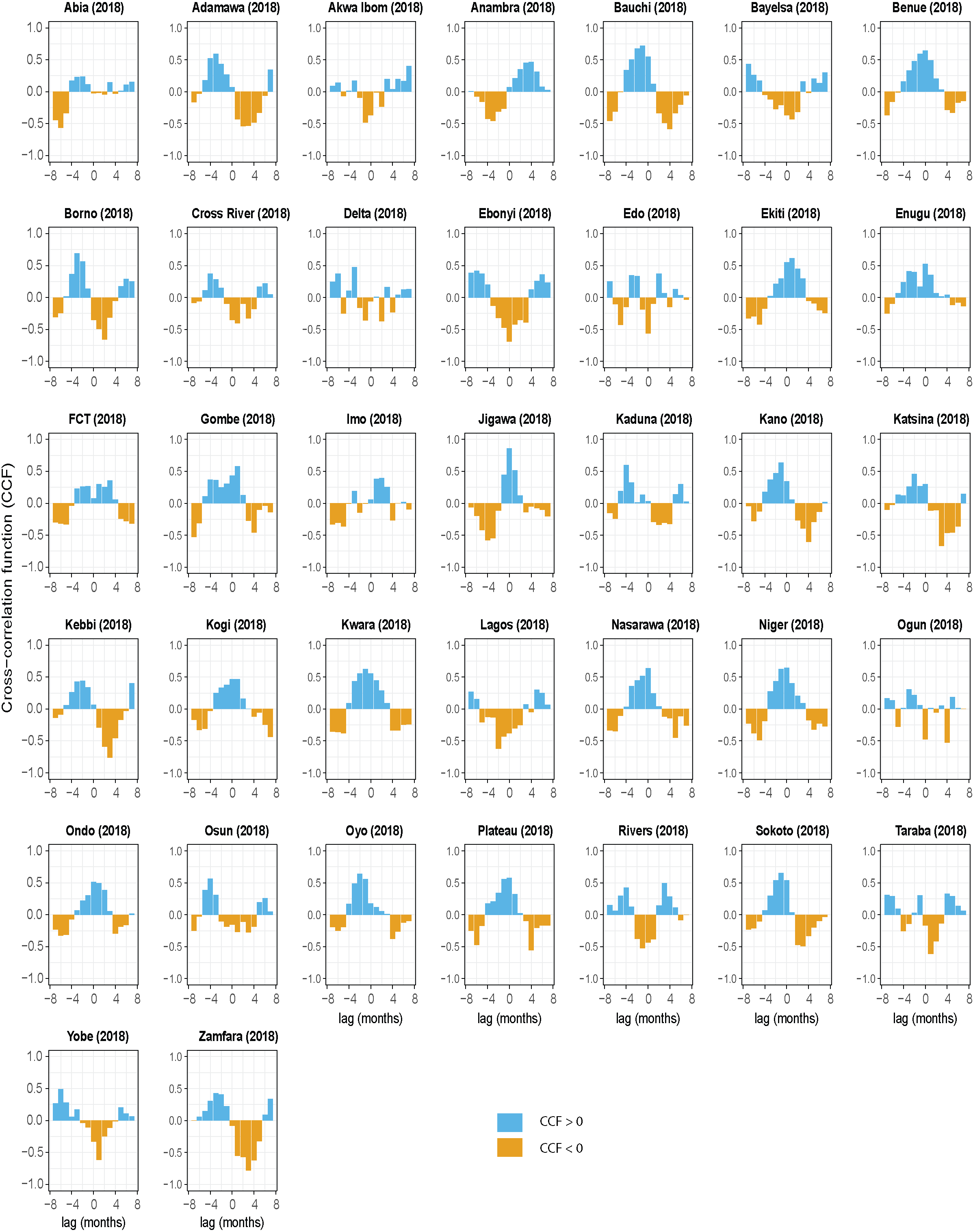


Figure S21: Comparison of DHIS2 and simulation seasonality trends in 2018 with a cross-correlation function (CCF). CCF at the time lag zero is a measure of the contemporaneous correlation or the linear relationship between the two time series.

**References**

1. Kaufman L, Rousseeuw PJ. Clustering large data sets. In: Gelsema ES, Kanal LN, editors. Pattern Recognit Pract [Internet]. Elsevier; 1986 [cited 2020 Aug 14]. p. 425–37. Available from: https://linkinghub.elsevier.com/retrieve/pii/B978044487877950039X

2. Kaufman L, Rousseeuw PJ. Finding Groups in Data [Internet]. Kaufman L, Rousseeuw PJ, editors. Find. Groups Data An Introd. to Clust. Anal. Hoboken, NJ, USA: John Wiley & Sons, Inc.; 1990 [cited 2020 Aug 18]. Available from: http://doi.wiley.com/10.1002/9780470316801

3. Maechler M, Rousseeuw P, Struyf A, Hubert M, Hornik K. cluster: Cluster Analysis Basics and Extensions. 2019.

4. Funk C, Nicholson SE, Landsfeld M, Klotter D, Peterson P, Harrison L. The Centennial Trends Greater Horn of Africa precipitation dataset. Sci Data [Internet]. Nature Publishing Groups; 2015 [cited 2020 Aug 14];2:1–17. Available from: www.nature.com/scientificdata

5. Weiss DJ, Bhatt S, Mappin B, Van Boeckel TP, Smith DL, Hay SI, et al. Air temperature suitability for Plasmodium falciparum malaria transmission in Africa 2000-2012: A high-resolution spatiotemporal prediction. Malar J [Internet]. BioMed Central Ltd.; 2014 [cited 2020 Jul 8];13. Available from: https://pubmed.ncbi.nlm.nih.gov/24886586/

6. Sinka ME, Golding N, Massey NC, Wiebe A, Huang Z, Hay SI, et al. Modelling the relative abundance of the primary African vectors of malaria before and after the implementation of indoor, insecticide-based vector control. Malar J [Internet]. BioMed Central; 2016 [cited 2020 Aug 14];15:142. Available from: https://malariajournal.biomedcentral.com/articles/10.1186/s12936-016-1187-8

7. Hay SI, Snow RW. The Malaria Atlas Project: Developing global maps of malaria risk [Internet]. PLoS Med. Public Library of Science; 2006 [cited 2020 Aug 14]. p. 2204–8. Available from: https://www.ncbi.nlm.nih.gov/pmc/articles/PMC1762059/

8. Gething PW, Patil AP, Smith DL, Guerra CA, Elyazar IRF, Johnston GL, et al. A new world malaria map: Plasmodium falciparum endemicity in 2010. Malar J [Internet]. BioMed Central; 2011 [cited 2020 Aug 14];10:378. Available from: /pmc/articles/PMC3274487/?report=abstract

9. Bertozzi-Villa A, Bever C, Koenker H, Weiss DJ, Vargas-Ruiz C, Nandi AK, et al. Maps and Metrics of Insecticide-Treated Net Coverage in Africa: Access, Use, and Nets-Per-Capita, 2000-2020. 2021.Available from https://www.researchsquare.com/article/rs-199628/v1.pdf

10. World Health Organization. World Malaria Report 2017 [Internet]. Geneva; 2017. Available from: https://apps.who.int/iris/bitstream/handle/10665/259492/9789241565523-eng.pdf;jsessionid=881CFD80B32ED1AF3A9D299643719B21?sequence=1

11. Hancock PA, Hendriks CJM, Tangena JA, Gibson H, Hemingway J, Coleman M, et al. Mapping trends in insecticide resistance phenotypes in African malaria vectors. PLoS Biol [Internet]. Public Library of Science; 2020 [cited 2020 Oct 2];18:e3000633. Available from: https://doi.org/10.1371/journal.pbio.3000633

12. Eckhoff P. Mathematical models of within-host and transmission dynamics to determine effects of malaria interventions in a variety of transmission settings. Am J Trop Med Hyg [Internet]. The American Society of Tropical Medicine and Hygiene; 2013 [cited 2020 Oct 2];88:817–27. Available from: /pmc/articles/PMC3752743/?report=abstract

13. Lengeler C. Insecticide-treated bed nets and curtains for preventing malaria. Cochrane Database Syst Rev [Internet]. Wiley; 2004 [cited 2020 Oct 2]; Available from: https://pubmed.ncbi.nlm.nih.gov/15106149/

14. Ngufor C, Tchicaya E, Koudou B, N’Fale S, Dabire R, Johnson P, et al. Combining organophosphate treated wall linings and long-lasting insecticidal nets for improved control of pyrethroid resistant Anopheles gambiae. PLoS One [Internet]. Public Library of Science; 2014 [cited 2021 Feb 18];9. Available from: https://pubmed.ncbi.nlm.nih.gov/24409286/

15. Badolo A, Guelbeogo WM, Tiono AB, Traoré A, Sagnon N, Sirima SB. Experimental hut evaluation of Fendona 6SC®-treated bednets and interceptor® long-lasting nets against Anopheles gambiae s.l. in Burkina Faso. J Vector Borne Dis. 2012;

16. Toe KH, Müller P, Badolo A, Traore A, Sagnon N, Dabiré RK, et al. Do bednets including piperonyl butoxide offer additional protection against populations of Anopheles gambiae s.l. that are highly resistant to pyrethroids? An experimental hut evaluation in Burkina Fasov. Med Vet Entomol. 2018;

17. Bayili K, N’do S, Namountougou M, Sanou R, Ouattara A, Dabiré RK, et al. Evaluation of efficacy of Interceptor® G2, a long-lasting insecticide net coated with a mixture of chlorfenapyr and alpha-cypermethrin, against pyrethroid resistant Anopheles gambiae s.l. in Burkina Faso. Malar J. 2017;

18. Churcher TS, Lissenden N, Griffin JT, Worrall E, Ranson H. The impact of pyrethroid resistance on the efficacy and effectiveness of bednets for malaria control in Africa. Elife. eLife Sciences Publications Ltd; 2016;5.

19. Tiono AB, Ouédraogo A, Ouattara D, Bougouma EC, Coulibaly S, Diarra A, et al. Efficacy of Olyset Duo, a bednet containing pyriproxyfen and permethrin, versus a permethrin-only net against clinical malaria in an area with highly pyrethroid-resistant vectors in rural Burkina Faso: a cluster-randomised controlled trial. Lancet. Lancet Publishing Group; 2018;392:569–80.

20. Menéndez C, Bardají A, Sigauque B, Romagosa C, Sanz S, Serra-Casas E, et al. A randomized placebo-controlled trial of intermittent preventive treatment in pregnant women in the context of insecticide treated nets delivered through the antenatal clinic. PLoS One [Internet]. Public Library of Science; 2008 [cited 2020 Nov 24];3. Available from: /pmc/articles/PMC2277457/?report=abstract

21. World Health Organisation. WHO Policy recommendation on Intermittent Preventive Treatment during infancy with sulphadoxine-pyrimethamine (SP-IPTi) for Plasmodium falciparum malaria control in Africa [Internet]. World Heal. Organ. 2010. Available from: https://www.who.int/malaria/news/WHO_policy_recommendation_IPTi_032010.pdf?ua=1

22. Esu EB, Oringanje C, Meremikwu MM. Intermittent preventive treatment for malaria in infants [Internet]. Cochrane Database Syst. Rev. John Wiley and Sons Ltd; 2019 [cited 2020 Dec 2]. Available from: /pmc/articles/PMC6887842/?report=abstract

23. Modiano D, Sirima BS, Sawadogo A, Sanou I, Paré J, Konaté A, et al. Severe malaria in Burkina Faso: Influence of age and transmission level on clinical presentation. Am J Trop Med Hyg [Internet]. American Society of Tropical Medicine and Hygiene; 1998 [cited 2021 Jan 12];59:539–42. Available from: https://pubmed.ncbi.nlm.nih.gov/9790426/

24. Olliaro P. Mortality associated with severe Plasmodium falciparum malaria increases with age [Internet]. Clin. Infect. Dis. Clin Infect Dis; 2008 [cited 2021 Jan 12]. p. 158–60. Available from: https://pubmed.ncbi.nlm.nih.gov/18564928/

25. Dondorp AM, Fanello CI, Hendriksen ICE, Gomes E, Seni A, Chhaganlal KD, et al. Artesunate versus quinine in the treatment of severe falciparum malaria in African children (AQUAMAT): an open-label, randomised trial. Lancet. Elsevier; 2010;376:1647–57.

26. Von Seidlein L, Olaosebikan R, Hendriksen ICE, Lee SJ, Adedoyin OT, Agbenyega T, et al. Predicting the clinical outcome of severe falciparum malaria in African children: Findings from a large randomized trial. Clin Infect Dis [Internet]. Clin Infect Dis; 2012 [cited 2021 Jan 7];54:1080–90. Available from: https://pubmed.ncbi.nlm.nih.gov/22412067/

27. Camponovo F, Bever CA, Galactionova K, Smith T, Penny MA. Incidence and admission rates for severe malaria and their impact on mortality in Africa. Malar J [Internet]. BioMed Central; 2017 [cited 2021 Jan 12];16:1–12. Available from: https://malariajournal.biomedcentral.com/articles/10.1186/s12936-016-1650-6

28. Group† TA-QMS. A meta-analysis using individual patient data of trials comparing artemether with quinine in the treatment of severe falciparum malaria. Trans R Soc Trop Med Hyg. 2001;95:637–50.

29. Thwing J, Eisele TP, Steketee RW. Protective efficacy of malaria case management for preventing malaria mortality in children: a systematic review for the Lives Saved Tool. BMC Public Health. 2011;11:S14.

30. Greenberg AE, Ntumbanzondo M, Ntula N, Mawa L, Howell J, Davachi F. Hospital-based surveillance of malaria-related paediatric morbidity and mortality in Kinshasa, Zaire. Bull World Health Organ [Internet]. World Health Organization; 1989 [cited 2021 Jan 12];67:189–96. Available from: /pmc/articles/PMC2491235/?report=abstract

31. Zucker JR, Ruebush TK, Obonyo C, Otieno J, Campbell CC. The mortality consequences of the continued use of chloroquine in Africa: Experience in Siaya, Western Kenya. Am J Trop Med Hyg. 2003;

32. Zucker JR, Lackritz EM, Ruebush TK, Hightower AW, Adungosi JE, Were JBO, et al. Childhood mortality during and after hospitalization in western Kenya: Effect of malaria treatment regimens. Am J Trop Med Hyg. 1996;

33. Ross A, Maire N, Molineaux L, Smith T. An epidemiologic model of severe morbidity and mortality caused by Plasmodium falciparum. Am J Trop Med Hyg. 2006;

34. Marsh K, Snow RW. Malaria transmission and morbidity. Parassitologia [Internet]. 1999 [cited 2021 Feb 16];41:241–6. Available from: https://europepmc.org/article/med/10697862

35. McCarthy KA, Wenger EA, Huynh GH, Eckhoff PA. Calibration of an intrahost malaria model and parameter ensemble evaluation of a pre-erythrocytic vaccine. Malar J [Internet]. BioMed Central Ltd.; 2015 [cited 2021 Feb 16];14:6. Available from: https://malariajournal.biomedcentral.com/articles/10.1186/1475-2875-14-6

36. Granja AC, Machungo F, Gomes A, Bergstrom S, Brabin B. Malaria related maternal mortality in Mozambique urban. Ann Trop Med Parasitol. 1998;92:257–63.

37. Schantz-Dunn J, Nour NM. Malaria and pregnancy: a global health perspective. Rev Obstet Gynecol. 2009;

38. Kalilani L, Mofolo I, Chaponda M, Rogerson SJ, Meshnick SR. The effect of timing and frequency of Plasmodium falciparum infection during pregnancy on the risk of low birth weight and maternal anemia. Trans R Soc Trop Med Hyg. 2010;

39. Huynh B-T, Fievet N, Gbaguidi G, Borgella S, Mévo BG, Massougbodji A, et al. Malaria associated symptoms in pregnant women followed-up in Benin. Malar J. 2011;10:72.

40. Brabin B. Malaria in pregnancy: current issues . African Heal [Internet]. 1997 [cited 2021 Jan 12];19. Available from: https://pubmed.ncbi.nlm.nih.gov/12292301/

41. Desai M, ter Kuile FO, Nosten F, McGready R, Asamoa K, Brabin B, et al. Epidemiology and burden of malaria in pregnancy [Internet]. Lancet Infect. Dis. Lancet Infect Dis; 2007 [cited 2021 Jan 12]. p. 93–104. Available from: https://pubmed.ncbi.nlm.nih.gov/17251080/

42. Guyatt H, Snow R. The epidemiology and burden of Plasmodium falciparum-related anemia among pregnant women in sub-Saharan Africa. Am J Trop Med Hyg. 2001;64:36–44.

43. Shulman CE, Marshall T, Dorman EK, Bulmer JN, Cutts F, Peshu N, et al. Malaria in pregnancy: Adverse effects on haemoglobin levels and birthweight in primigravidae and multigravidae. Trop Med Int Heal [Internet]. Trop Med Int Health; 2001 [cited 2021 Jan 12];6:770–8. Available from: https://pubmed.ncbi.nlm.nih.gov/11679125/

44. Shulman CE, Dorman EK, Cutts F, Kawuondo K, Bulmer JN, Peshu N, et al. Intermittent sulphadoxine-pyrimethamine to prevent severe anaemia secondary to malaria in pregnancy: a randomised placebo-controlled trial. Lancet. 1999;353:632–6.

45. Asa OO, Onayade AA, Fatusi AO, Ijadunola KT, Abiona TC. Efficacy of Intermittent Preventive Treatment of Malaria with Sulphadoxine-pyrimethamine in Preventing Anaemia in Pregnancy among Nigerian Women. Matern Child Health J. 2008;12:692–8.

46. Central Intelligence Agency. Nigeria. World Factb. 2021.

47. Sirima SB, Cotte AH, Konaté A, Moran AC, Asamoa K, Bougouma EC, et al. Malaria prevention during pregnancy: Assessing the disease burden one year after implementing a program of intermittent preventive treatment in Koupéla District, Burkina Faso. Am J Trop Med Hyg. 2006;

48. Rogerson SJ. Management of malaria in pregnancy. Indian J. Med. Res. 2017.

49. Luxemburger C, McGready R, Kham A, Morison L, Cho T, Chongsuphajaisiddhi T, et al. Effects of malaria during pregnancy on infant mortality in an area of low malaria transmission. Am J Epidemiol. 2001;

50. Steketee RW, Nahlen BL, Parise ME, Menendez C. The burden of malaria in pregnancy in malaria-endemic areas. Am J Trop Med Hyg. 2001.

51. Valea I, Tinto H, Drabo MK, Huybregts L, Sorgho H, Ouedraogo J-B, et al. An analysis of timing and frequency of malaria infection during pregnancy in relation to the risk of low birth weight, anaemia and perinatal mortality in Burkina Faso. Malar J. 2012;11:71.

52. Bardají A, Sigauque B, Sanz S, Maixenchs M, Ordi J, Aponte JJ, et al. Impact of Malaria at the End of Pregnancy on Infant Mortality and Morbidity. J Infect Dis. 2011;203:691–9.

53. Menéndez C, Bardají A, Sigauque B, Sanz S, Aponte JJ, Mabunda S, et al. Malaria prevention with IPTp during pregnancy reduces neonatal mortality. PLoS One. 2010;

54. He Z, Bishwajit G, Yaya S, Cheng Z, Zou D, Zhou Y. Prevalence of low birth weight and its association with maternal body weight status in selected countries in Africa: A cross-sectional study. BMJ Open. 2018;

55. Central Intelligence Agency. Burkina Faso [Internet]. World Factb. 2017. Available from: https://www.cia.gov/the-world-factbook/countries/burkina-faso/#people-and-society

56. Kayentao K, Garner P, Maria van Eijk A, Naidoo I, Roper C, Mulokozi A, et al. Intermittent Preventive Therapy for Malaria During Pregnancy Using 2 vs 3 or More Doses of Sulfadoxine-Pyrimethamine and Risk of Low Birth Weight in Africa: Systematic Review and Meta-analysis. JAMA. 2013;309:594–604.
